# Targeting Viperin prevents coxsackievirus B3-induced acute heart failure

**DOI:** 10.1038/s41421-025-00778-0

**Published:** 2025-04-08

**Authors:** Yukang Yuan, Liping Qian, Ying Miao, Qun Cui, Ting Cao, Yong Yu, Tingting Zhang, Qian Zhao, Renxia Zhang, Tengfei Ren, Yibo Zuo, Qian Du, Caixia Qiao, Qiuyu Wu, Zhijin Zheng, Minqi Li, Y. Eugene Chinn, Wei Xu, Tianqing Peng, Ruizhen Chen, Sidong Xiong, Hui Zheng

**Affiliations:** 1 The First Affiliated Hospital of Soochow University, Institutes of Biology and Medical Sciences, Suzhou Medical College, Soochow University, Suzhou, Jiangsu China; 2 International Institute of Infection and Immunity, Institutes of Biology and Medical Sciences, Soochow University, Suzhou, Jiangsu China; 3 Jiangsu Key Laboratory of Infection and Immunity, Soochow University, Suzhou, Jiangsu China; 4 Department of Laboratory Medicine, Institute of Laboratory Medicine, Sichuan Provincial People’s Hospital, School of Medicine, University of Electronic Science and Technology of China, Chengdu, Sichuan China; 5 Institute for Cardiovascular Science, Collaborative Innovation Center of Hematology, Soochow University, Suzhou, Jiangsu China; 6 Department of Cardiology, Shanghai Institute of Cardiovascular Diseases, Zhongshan Hospital, Fudan University, Shanghai, China; 7 Medical College of Nantong University, Nantong, Jiangsu China; 8 Lawson Health Research Institute, London Health Sciences Centre, London, ON Canada; 9 Department of Medicine, Department of Pathology and Laboratory Medicine, Western University, London, ON Canada

**Keywords:** Mechanisms of disease, Cell signalling, Ubiquitylation

## Abstract

Coxsackievirus B3 (CVB3)-induced acute heart failure (AHF) is a common cause of cardiogenic death in young- and middle-aged people. However, the key molecular events linking CVB3 to AHF remain largely unknown, resulting in a lack of targeted therapy strategies thus far. Here, we unexpectedly found that Viperin deficiency does not promote CVB3 infection but protects mice from CVB3-induced AHF. Importantly, cardiac-specific expression of Viperin can induce cardiac dysfunction. Mechanistically, CVB3-encoded 3C protease rescues Viperin protein expression in cardiomyocytes by lowering UBE4A. Viperin in turn interacts with and reduces STAT1 to activate SGK1-KCNQ1 signaling, and eventually leads to cardiac electrical dysfunction and subsequent AHF. Furthermore, we designed an interfering peptide VS-IP1, which blocked Viperin-mediated STAT1 degradation and therefore prevented CVB3-induced AHF. This study established the first signaling link between CVB3 and cardiac electrical dysfunction, and revealed the potential of interfering peptides targeting Viperin for the treatment of CVB3-induced AHF.

## Introduction

Heart failure (HF) affects approximately 23 million people worldwide, and brings a growing public health burden due to high mortality^1,2^. HF is a progressive clinical syndrome caused by functional and/or structural cardiac abnormalities^3^. An important hallmark of HF is a decrease in myocardial contractility, which usually results in reduced left ventricular function to varying degrees, including reduced left ventricular ejection fraction (LVEF) and left ventricular fractional shortening (LVFS)^3–5^. In addition, serum brain-type natriuretic peptide (BNP) is a commonly used gold-standard marker for the diagnosis of acute HF (AHF) in clinical practice^6,7^.

Recent studies have identified several important molecules that regulate chronic HF (CHF) induced by different stimuli. For example, the Hippo pathway, MARK4, and Phosphodiesterase 4D have been reported to play essential roles in ischaemic HF^8–10^. SERCA2a, BET bromodomain protein, and mitochondrial DNA regulate transverse aortic constriction (TAC)-induced HF^11–13^. In addition, beta2-adrenergic receptor redistribution can affect CHF^14^. Despite good advances from these studies, as well as other studies, most studies focused on CHF. The crucial molecular events that mediate AHF remain largely unknown.

Coxsackievirus B3 (CVB3) is a common pathogen that induces both AHF and CHF^15^. The incidence rate of CVB3-induced heart diseases has gradually increased in the past two decades worldwide. Severe CVB3 infection could cause AHF, which is a common cause of sudden cardiac death in both young- and middle-aged people. To date, some studies have demonstrated that viral myocarditis, which is mediated by both CVB3-induced damage of cardiomyocytes and host-cell inflammatory responses, is an important promoting factor for the onset of HF^16–18^. Thus, much progress has been achieved in exploring the regulation of CVB3-induced myocarditis^17,19,20^. However, CVB3 is not the only virus that can infect the heart and cause inflammatory responses, but CVB3 is more likely to induce AHF than other viruses, suggesting that CVB3 could execute certain specific actions. Of note, it has been well documented that CVB3 can induce electrical cardiac abnormalities, which progressively lead to functional cardiac abnormalities and even AHF^18,21,22^. Unlike CVB3-induced CHF, which is generally involved in structural cardiac changes, CVB3-induced AHF usually does not result in remarkable structural changes due to the rapid onset and could only lead to functional cardiac abnormalities. However, how CVB3 specifically regulates functional cardiac abnormalities to trigger AHF is poorly understood. In this study, we revealed a crucial mechanism by which CVB3 induces Viperin to mediate cardiac dysfunction and AHF.

## Results

### Viperin is crucial for mediating CVB3-induced cardiac dysfunction and AHF

**Fig. 1:**
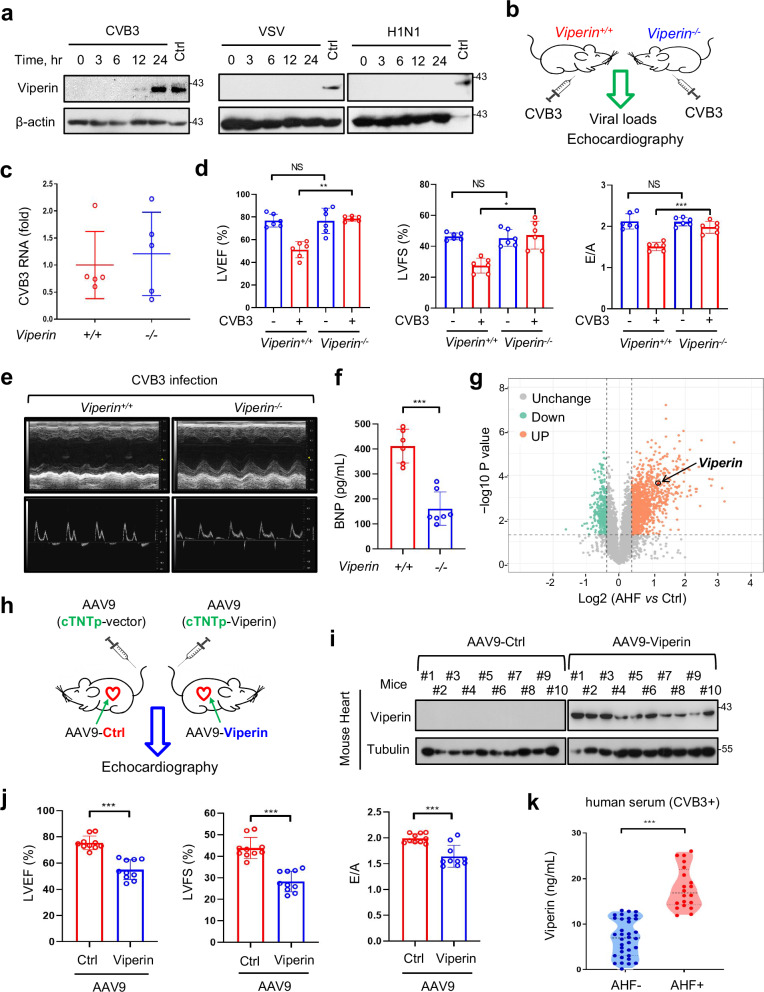
Viperin is crucial for mediating CVB3-induced cardiac dysfunction and AHF. **a** Western blot analysis of Viperin expression in HL-1 cells infected with CVB3 (MOI = 10), VSV (MOI = 10) or H1N1 (MOI = 10) for different time periods. The controls (Ctrl) are whole cell lysates from MEF cells treated with IFNβ. **b**, **c** *Viperin*^−/−^ mice (*n* = 5) and their littermate controls (*Viperin*^+/+^) (*n* = 5) were infected with CVB3 (2 × 10^5^ PFU per gram body, intraperitoneally (i.p.)) for 7 days (**b**). The *CVB3* RNA levels in mouse heart were analyzed by Real-time quantitative PCR (RT-qPCR) (**c**). **d**–**f** *Viperin*^−/−^ mice (*n* = 6) and their littermate controls (*Viperin*^+/+^) (*n* = 6) were infected with or without CVB3 (2 × 10^5^ PFU per gram body, i.p.) for 7 days. The LVEF, LVFS, and E/A ratio in *Viperin*^−/−^ and *Viperin*^+/+^ mice were analyzed by echocardiography (**d**). The representative images of the echocardiography were shown (**e**). Serum BNP levels were analyzed by ELISA (**f**). **g** Three CVB3-infected mice with LVEF below 50% and LVFS below 20% were selected as the AHF group according to the literature, and three CVB3-infected mice with LVEF above 50% and LVFS above 20% were selected as the control (Ctrl) group. The hearts of Ctrl (*n* = 3) and AHF (*n* = 3) mice were subjected to a quantitative proteomic analysis with a TMT2-plex method. Differentially expressed proteins were analyzed in the volcano plot. **h** AAV9-Ctrl and AAV9-Viperin with the promoter *cTNTp* was injected into the mice. After 14 days, the mice were observed by echocardiography. **i** Viperin protein levels in AAV9-Ctrl mice (*n* = 10) and AAV9-Viperin mice (*n* = 10) heart tissues from **h** were analyzed by western blot assay. **j** The LVEF, LVFS, and E/A ratio in AAV9-Ctrl mice (*n* = 10) and AAV9-Viperin mice (*n* = 10) were analyzed by echocardiography. **k** ELISA analysis of serum Viperin protein levels in CVB3-positive patients without AHF (AHF^–^, *n* = 34) or CVB3-positive patients with AHF (AHF^+^, *n* = 20). *P < 0.05, **P < 0.01 and ***P < 0.001 (two-tailed unpaired Student’s *t*-test). The graphs show the means ± SEM for five to six (**c**, **d**, **f**) and ten (**j**) individual mice, or for individual persons (**k**). Data are representative of three independent experiments (**a**, **i**).

Our previous studies revealed that the antiviral molecule, Viperin (mouse ortholog, Rsad2) protein, can be produced only in very limited types of cells, such as macrophages and fibroblasts, during viral infection and even interferon (IFN) treatment^23^. For example, vesicular stomatitis virus (VSV) infection cannot induce Viperin protein production in mouse lung, liver and kidney tissues, as well as heart tissues^23^. To our surprise, we noticed that Viperin protein can be produced in cardiomyocytes upon CVB3 infection (Fig. 1a). However, cardiomyocytes cannot produce Viperin protein when infected with VSV, influenza A virus (H1N1), Sendai virus (SeV) and herpes simplex virus (HSV) (Fig. 1a and Supplementary Fig. S1a). Thus, we sought to determine the significance of the Viperin protein induced by CVB3 infection in cardiomyocytes. Given that we have *Viperin*-knockout (*Viperin*^−/^^−^) mice in hand^24^, we employed the *Viperin*^−/−^ mice to observe the influence of Viperin on the disease progression of CVB3 infection (Fig. 1b). Interestingly, we found that there were no significant differences in CVB3 virus titers in serum between *Viperin*^*+/+*^ and *Viperin*^−/−^ mice on day 3 post infection (Supplementary Fig. S1b). Consistently, CVB3 viral levels in *Viperin*^*+/+*^ and *Viperin*^−/^^−^ mouse hearts were comparable on day 7 after CVB3 infection (Fig. 1c). Given that Viperin is a broad-spectrum antiviral protein, the observation that Viperin does not significantly affect in vivo CVB3 infection suggests that Viperin could have more complex regulation of CVB3 infection.

Next, we analyzed the potential effects of Viperin on mouse myocardial function. To this end, echocardiographic analysis was performed to determine mouse LVEF and LVFS, which are two important hallmarks of HF reflecting myocardial contractility. The results showed that normal *Viperin*^*+/+*^ mice without CVB3 infection had LVEF within the range of 70%‒80% and LVFS within the range of 40%‒50% (Supplementary Fig. S1c), while *Viperin*^*+/+*^ mice on day 7 after CVB3 infection had reduced LVEF (less than 60% on average) and reduced LVFS (less than 30% on average) (Fig. 1d), suggesting the impairment of myocardial function to some extent. We noticed that some of CVB3-infected *Viperin*^*+/+*^ mice had dramatically reduced LVEF (40%‒50%) and LVFS (approximately 20%) (Fig. 1d), which are similar to those reported LVEF and LVFS levels in HF mice in the literature^12,13^. Compared to *Viperin*^*+/+*^ mice, *Viperin*^−^^/−^ mice with CVB3 infection had significantly higher LVEF and LVFS, which were close to those of normal mice without CVB3 infection, demonstrating that *Viperin*^−/−^ mice had much better cardiac function (Fig. 1d, e). At the same time, the pulsed wave Doppler measurements of maximal early (E) and late (A) transmitral velocities in diastole were analyzed at mitral valve inflow. The results showed that during CVB3 infection, *Viperin*^−/−^ mice had a higher E/A ratio than *Viperin*^*+/+*^ mice (Fig. 1d, right). These data demonstrated that CVB3-induced Viperin is involved in impairing myocardial contractility that is commonly reflective of HF.

To confirm the effect of Viperin on AHF, we further analyzed another golden marker for the diagnosis of AHF, serum BNP. We noticed that the serum BNP levels of *Viperin*^*+/+*^ mice with CVB3 infection were greatly upregulated (300‒500 pg/mL), while most of *Viperin*^−^^/^^−^ mice had only slightly increased serum BNP levels (100‒130 pg/mL) (Fig. 1f). It has been reported that a concentration of 150 pg/mL shows an 83% positive predictive value and 84% accuracy for HF diagnosis in humans^25,26^, while the concentrations of serum BNP in humans and mice are actually comparable and generally below 100 pg/mL under normal conditions (Supplementary Fig. S1d). These AHF-related parameters demonstrated that CVB3 infection progressively induces AHF in *Viperin*^*+/+*^ mice, while *Viperin* knockout largely protects the mice against CVB3-induced AHF.

Consistent with Viperin protein production in CVB3-infected HL-1 cells, CVB3 infection induced *Viperin* mRNA expression in mouse primary cardiomyocytes in a time-dependent manner (Supplementary Fig. S1e). Next, we selected three CVB3-infected mice with LVEF below 50% and LVFS below 20% as the AHF group, which is equivalent to the LVEF and LVFS levels for mouse HF commonly used in the literature^12,13^. Then, we found by a quantitative proteomic analysis that compared to three control mice with LVEF above 50% and LVFS above 20%, the Viperin protein levels in the AHF group were higher than those in the control group (Fig. 1g). These results suggest that Viperin protein in hearts is induced by CVB3 infection, while high levels of Viperin protein in hearts could be correlated with damaged myocardial function. To further observe the effect of Viperin itself on myocardial contractility, we employed a mouse model with cardiac-specific Viperin expression, which used an Viperin construct in an AAV9 expression vector with the cardiomyocyte-specific promoter cTNTp^27^ (Fig. 1h). On day 14 post-injection with AAV9-Viperin, we observed that Viperin proteins were mainly expressed in mouse heart tissues (Fig. 1i and Supplementary Fig. S2a). Intriguingly, we observed that the LVEF, LVFS, and E/A ratio in many of Viperin-overexpressing mice were reduced to levels similar to those in CVB3-induced AHF mice (Fig. 1j and Supplementary Fig. S2b), suggesting that cardiac-specific overexpression of Viperin but not the controls in mice was able to induce moderate to severe cardiac dysfunction. Moreover, given that AHF results in the release of many intracellular proteins from the damaged cardiomyocytes, we collected human sera from 54 of CVB3-positive patients with or without AHF to determine Viperin protein levels. The results showed that although all patients were CVB3 positive, Viperin protein levels in the patients with AHF were significantly higher than those in the control patients without AHF (Fig. 1k), indicating the correlation between high levels of Viperin protein and AHF. In conjugating with the findings showing that *Viperin* knockout protects against CVB3-induced AHF, we think that Viperin is crucial for mediating cardiac dysfunction and AHF induced by CVB3.

### Viperin promotes SGK1 signaling in myocardial cells

**Fig. 2:**
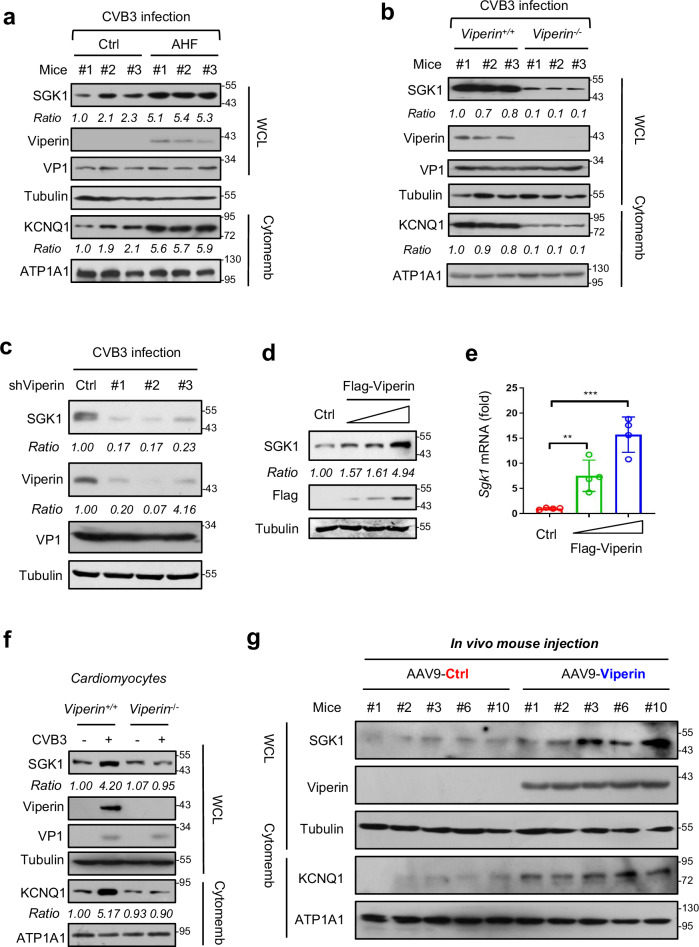
Viperin promotes SGK1 expression in myocardial cells. **a** Western blot analysis of SGK1, Viperin and cytomembrane KCNQ1 levels in the heart tissues of the Ctrl (*n* = 3) and AHF (*n* = 3) mice from the experiment of Fig. 1g. **b** *Viperin*^*+/+*^ (*n* = 3) and *Viperin*^−/−^ (*n* = 3) mice were infected with CVB3 (2 × 10^5^ PFU per gram body, i.p.) for 7 days. Western blot analysis was used to analyze SGK1 and cytomembrane KCNQ1 levels in mouse heart tissues. **c** Western blot analysis of SGK1 levels in HL-1 cells transfected with control shRNAs (Ctrl) or shRNAs against Viperin (shViperin, #1, #2, and #3) and then infected with CVB3 (MOI = 10) for 12 h. **d**, **e** Western blot analysis of SGK1 protein (**d**) or RT-qPCR analysis of *Sgk1* mRNA (**e**) in HL-1 cells transfected with increasing amounts of Flag-Viperin. **f** Western blot analysis of SGK1 levels in *Viperin*^*+/+*^ or *Viperin*^−/−^ HL-1 cells infected with CVB3 (MOI = 10) for 12 h. **g** The heart tissues that have relatively high levels of Viperin from experiment in Fig. 1I were selected. Western blot analysis was used to analyze SGK1 and cytomembrane KCNQ1 levels in mouse hearts (*n* = 5). **P < 0.01 and ***P < 0.001 (two-tailed unpaired Student’s *t*-test). Data are shown as means ± SD of four biological replicates (**e**), or are representative of three independent experiments (**a**‒**d**, **f**, **g**).

To date, the key molecules that mediate AHF during CVB3 infection remain largely unexplored. Current studies have demonstrated that HF is associated with altered electric properties of cardiomyocytes^28–30^. Interestingly, important research progress has demonstrated that serum- and glucocorticoid-regulated kinase-1 (SGK1) is persistently activated during HF, and SGK1 is both necessary and sufficient to induce hallmarks of electric and mechanical remodeling seen in HF^29^. More importantly, cardiac-specific expression of constitutively active SGK1 can induce cardiac dysfunction, such as reduced LVFS, while SGK1 inhibition protects against TAC-induced HF^29^. In line with these advances, we found that SGK1 protein levels were upregulated in heart tissues of *Viperin*^*+/+*^ mice with AHF (Fig. 2a). However, on day 7 after CVB3 infection, the heart tissues of *Viperin*^−^^/−^ mice had remarkably lower levels of SGK1 than those of *Viperin*^*+/+*^ mice (Fig. 2b), suggesting a positive correlation between Viperin and SGK1.

It has been reported that SGK1 signaling activates various types of cardiac ion channels associated with cardiac dysfunction^29,31,32^. Importantly, SGK1 can promote cytomembrane delivery of the potassium channel KCNQ1^31^. Thus, we detected KCNQ1 levels in cell membranes. Consistently, the heart tissues from *Viperin*^*+/+*^ mice with AHF had higher levels of cytomembrane KCNQ1 than those from the control mice (Fig. 2a). In comparison, the cytomembrane KCNQ1 levels in *Viperin*^−/−^ mice were lower than those in *Viperin*^*+/+*^ mice (Fig. 2b). Next, we examined whether Viperin regulates SGK1 expression in cardiomyocytes. The results showed that Viperin knockdown decreased SGK1 expression in cardiomyocytes during CVB3 infection (Fig. 2c), while Viperin overexpression upregulated the levels of both SGK1 protein (Fig. 2d) and *Sgk1* mRNA (Fig. 2e). Moreover, CVB3 infection increased SGK1 levels in cardiomyocytes, while in *Viperin*^−^^/−^ cells CVB3 infection cannot upregulate SGK1 levels any longer (Fig. 2f). Importantly, cardiac-specific overexpression of Viperin in mice resulted in higher levels of both SGK1 and cytomembrane KCNQ1 in mouse hearts (Fig. 2g), suggesting that Viperin promotes in vivo SGK1-KCNQ1 signaling in cardiomyocytes. Collectively, these findings suggested that Viperin activates the expression of both SGK1 and subsequent cardiac ion channels, thus mediating cardiac electrical dysfunction and AHF.

### Viperin regulates SGK1 expression by lowering STAT1 levels

**Fig. 3:**
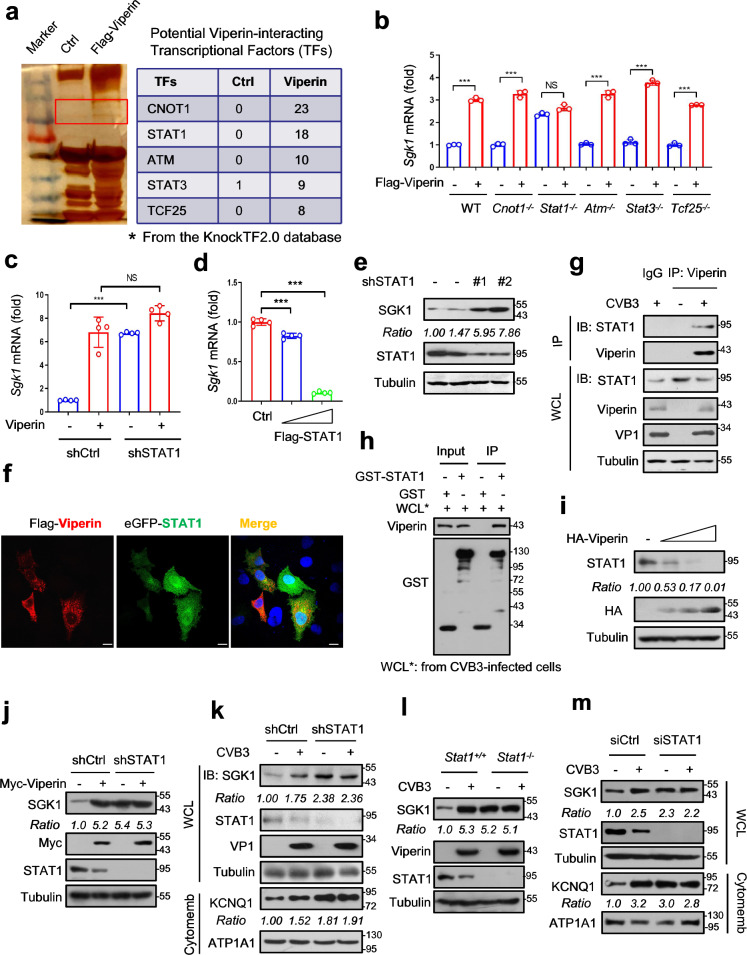
Viperin regulates SGK1 expression by lowering STAT1 levels. **a** Whole cell lysates from HEK293T cells transfected with Flag-Viperin were subjected to immunoprecipitation with Flag agarose (left). Mass spectrometry analysis of potential Viperin-binding TFs was shown (right). The predicted TFs of SGK1 were from the KnockTF2.0 database. **b** RT-qPCR analysis of *Sgk1* mRNA in HEK293T cells (WT or knockout of the indicated TFs) transfected with Flag-Viperin. **c** RT-qPCR analysis of *Sgk1* mRNA in HL-1 cells transfected with control shRNAs (shCtrl) or shRNAs against STAT1 (shSTAT1), together with or without Flag-Viperin. **d** RT-qPCR analysis of *Sgk1* mRNA in HL-1 cells transfected with increasing amounts of Flag-STAT1. **e** Western blot analysis of SGK1 levels in HL-1 cells transfected with shCtrl (‒) or shSTAT1 (#1 and #2). **f** Confocal microscopy in HeLa cells transfected with Flag-Viperin and eGFP-STAT1. Scale bar, 5 μm. **g** Immunoprecipitation analysis of the interaction between Viperin and STAT1 in HL-1 cells infected with CVB3 (MOI = 10) for 12 h. **h** GST pulldown assay was performed to analyze the interaction between Viperin and STAT1. **i** Western blot analysis of STAT1 levels in HL-1 cells transfected with increasing amounts of HA-Viperin. **j** Western blot analysis of SGK1 levels in HL-1 cells transfected with shCtrl or shSTAT1, together with or without Flag-Viperin. **k** Western blot analysis of SGK1 levels in HL-1 cells transfected with shCtrl or shSTAT1, followed by CVB3 infection (MOI = 10) for 12 h. **l** Western blot analysis of SGK1 and Viperin levels in *Stat1*^*+/+*^ or *Stat1*^−/−^ cells infected with or without CVB3 (MOI = 10). **m** Mice were injected the siCtrl or siSTAT1 in the tail vein at a dose of 0.1 mg per mouse once a day for four consecutive days, and then were infected with or without CVB3 (2 × 10^5^ PFU per gram body, i.p.) for 7 days. Western blot analysis was used to analyze SGK1, STAT1 and cytomembrane KCNQ1 levels. NS not significant (*P* > 0.05), ***P < 0.001, two-tailed unpaired Student’s *t*-test (**b**, **c**), one-way analysis of variance (ANOVA) (**d**). Data are shown as means ± SD of four biological replicates (**b**‒**d**) or are representative of three independent experiments (**e**, **g**‒**m**).

Given that Viperin is not a transcriptional factor (TF) but upregulates *Sgk1* mRNA levels, we hypothesized that certain Viperin-interacting transcriptional factors could contribute to *Sgk1* mRNA upregulation. Thus, Flag-Viperin-interacting proteins were immunoprecipitated from cells transfected with Flag-Viperin and then were subjected to a mass spectrometry analysis. We noticed several putative TFs that could interact with Viperin (Fig. 3a). Among them, STAT1 was predicted by the KnockTF2.0 database to be a TF that could inhibit *SGK1* expression. We found that knockout of *STAT1* but not other TFs significantly promoted *SGK1* transcriptional expression (Fig. 3b). Importantly, in *STAT1*-knockout cells, Viperin could no longer upregulate *Sgk1* mRNA expression (Fig. 3b). Similarly, knockdown of STAT1 upregulated *Sgk1* mRNA levels (Fig. 3c and Supplementary Fig. S3a), while STAT1 overexpression inhibited *Sgk1* mRNA expression (Fig. 3d). Consistently, STAT1 knockdown upregulated SGK1 protein levels in cardiomyocytes (Fig. 3e). Collectively, these findings demonstrated that STAT1 is an inhibitory regulator of SGK1 expression.

Further studies revealed that Flag-Viperin can co-localize with eGFP-STAT1 (Fig. 3f). In addition, both CVB3-induced and IFN-induced Viperin can interact with endogenous STAT1 (Fig. 3g and Supplementary Fig. S3b), while another IFN-induced protein (IFIT1) cannot interact with STAT1 in HL-1 cells infected with CVB3 (Supplementary Fig. S3c). Furthermore, GST pulldown assay confirmed the interaction between Viperin and STAT1 (Fig. 3h). Interestingly, we noticed that the cells with Viperin expression had reduced STAT1 protein levels (Fig. 3g). In addition, Viperin overexpression did not affect *Stat1* mRNA levels (Supplementary Fig. S3d) but downregulated cellular STAT1 protein levels in various types of cells (Fig. 3i and Supplementary Fig. S3e). These data suggest that Viperin negatively regulates STAT1 protein levels. In addition, deletion of the SAM domain of Viperin restricted its interaction with STAT1 (Supplementary Fig. S3f), indicating that STAT1 could bind to the SAM domain of Viperin. In line with this observation, deletion of the SAM domain of Viperin abolished the ability of Viperin to downregulate STAT1 protein levels (Supplementary Fig. S3g). Based on the above findings, we speculated that Viperin can downregulate STAT1 and in turn promote *Sgk1* transcriptional expression. Consistent with this speculation, both Viperin overexpression and CVB3 infection can no longer upregulate SGK1 protein levels in STAT1-deficient cells (Fig. 3j, k). Together, these findings suggest that CVB3-induced Viperin protein lowers cellular STAT1 to enhance SGK1 expression.

We found that CVB3 downregulated STAT1 levels in both HL-1 and mouse primary cardiomyocytes (Supplementary Fig. S3h, i), while knockout of *Viperin* abolished CVB3-induced STAT1 downregulation and SGK1 signaling upregulation (Supplementary Fig. S3j, k). Moreover, we found that STAT1 deficiency did not affect CVB3-induced Viperin expression but abolished CVB3-induced SGK1 expression during CVB3 infection (Fig. 3l, m). These findings suggest that STAT1 functions as a downstream effector of Viperin and as an upstream factor of SGK1 during CVB3 infection. Furthermore, we found that *SGK1* knockout did not affect CVB3-induced STAT1 downregulation (Supplementary Fig. S3l), suggesting that STAT1 is the upstream of SGK1 during CVB3 infection. In conjunction with the previous findings showing that CVB3 infection resulted in STAT1 downregulation and SGK1 upregulation, these findings suggested that SGK1 functions downstream of STAT1 during CVB3 infection.

### Viperin promotes STAT1 ubiquitination and degradation via UBR5

**Fig. 4:**
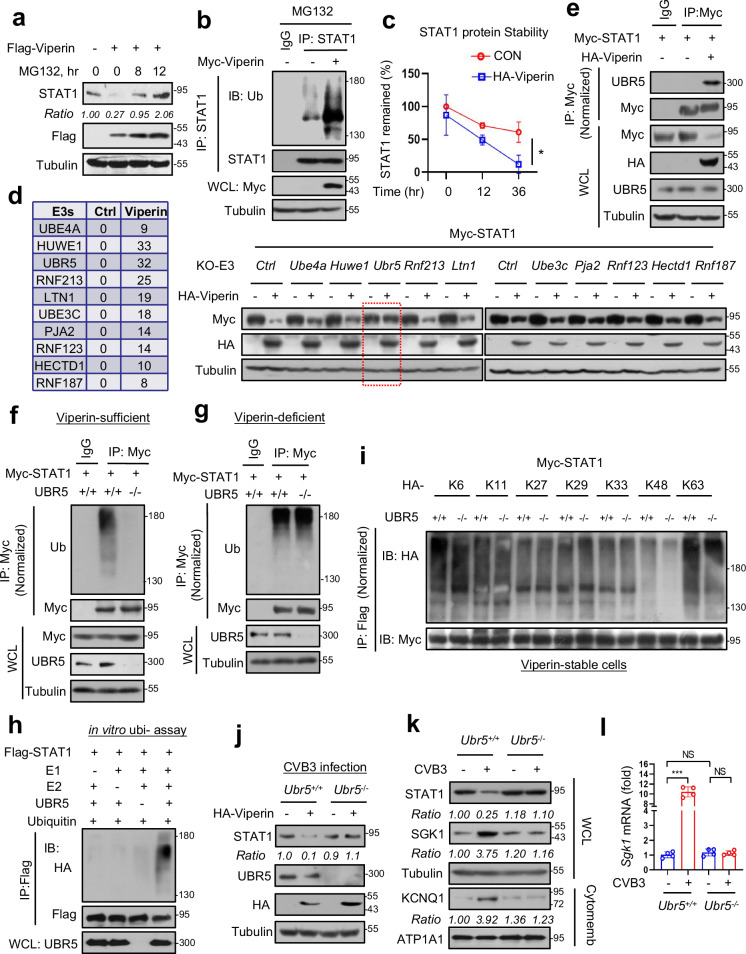
Viperin promotes STAT1 ubiquitination and degradation dependently on UBR5. **a** Western blot analysis of STAT1 levels in HEK293T cells transfected with Flag-Viperin and treated with MG132 (10 μM) as indicated. **b** Immunoprecipitation analysis of STAT1 ubiquitination in HL-1 cells transfected with or without Myc-Viperin and then treated with MG132 (10 μM, 4 h). **c** cycloheximide (CHX) pulse chase assay was used to analyze Flag-STAT1 protein stability in HEK293T cells co-transfected with Flag-STAT1 and HA-Viperin, followed by CHX treatment (50 μM) for 12 h and 36 h. **d** Mass spectrometry analysis of Viperin-binding proteins in HEK293T cells transfected with Flag-Viperin. The putative ubiquitin E3 ligases interacting with Viperin are listed. Western blot was used to analyze Myc-STAT1 levels in cells with knockout of these ubiquitin E3 ligases that were transfected with or without HA-Viperin. **e** Immunoprecipitation analysis of the interaction between UBR5 and STAT1 in HL-1 cells transfected with or without HA-Viperin. **f**, **g** *Ubr5*^*+/+*^ or *Ubr5*^−/−^ cells were made by CRISPR-Cas9 in both stable Viperin-expressing HEK293T cells (**f**) and intact HEK293T cells (Viperin protein-deficient) (**g**). Immunoprecipitation was used to analyze STAT1 ubiquitination. **h** In vitro ubiquitination assay for analysis of Flag-STAT1 ubiquitination by the recombinant UBR5 proteins. Flag-STAT1 was immunoprecipitated by Flag beads. Flag-STAT1 ubiquitination was determined using an anti-Flag antibody using western blot analysis. **i** Immunoprecipitation analysis of STAT1 ubiquitination types in stable Viperin-expressing HEK293T cells cotransfected with Myc-STAT1 and different types of HA-Ub. **j** Western blot analysis of STAT1 levels in *Ubr5*^*+/+*^ or *Ubr5*^−/−^ cells transfected with HA-Viperin. **k** Western blot analysis of STAT1, SGK1 and cytomembrane KCNQ1 levels in *Ubr5*^*+/+*^ or *Ubr5*^−/−^ HL-1 cells infected with or without CVB3 (MOI = 10) for 12 h. **l** RT-qPCR analysis of *Sgk1* mRNA in *Ubr5*^*+/+*^ or *Ubr5*^−/−^ HL-1 cells infected with or without CVB3 (MOI = 10) for 12 h. NS, not significant (*P* > 0.05), ***P < 0.001, two-tailed unpaired Student’s *t*-test. Data are shown as means ± SD of four biological replicates (**l**) or are representative of three independent experiments (**a**, **b**, **d**‒**k**).

Given that Viperin can downregulate exogenously expressed Flag-STAT1 (Supplementary Fig. S3g), we next determined whether Viperin could regulate STAT1 at the protein level. We found that a proteasome inhibitor, MG132, inhibited Viperin-mediated downregulation of STAT1 protein levels (Fig. 4a). Viperin overexpression promoted STAT1 ubiquitination in both HL-1 and 293 T cells (Fig. 4b and Supplementary Fig. S4a), while Viperin knockdown reduced STAT1 ubiquitination (Supplementary Fig. S4b). In addition, CVB3 infection also upregulated the ubiquitination levels of STAT1 (Supplementary Fig. S4c). In line with the upregulation of STAT1 ubiquitination, Viperin overexpression lowered STAT1 protein stability in cardiomyocytes (Fig. 4c and Supplementary Fig. S4d). These data demonstrated that Viperin regulates STAT1 ubiquitination and protein stability.

To identify the potential ubiquitin E3 ligases that contribute to STAT1 ubiquitination induced by Viperin, we first analyzed those defined or putative Viperin-interacting ubiquitin E3 ligases identified in our previous studies^23^. We noticed that compared to the other ubiquitin E3 ligases, *UBR5* knockout largely blocked Viperin-mediated downregulation of STAT1 (Fig. 4d). Interestingly, it was very difficult to observe the interaction between STAT1 and UBR5 in Viperin protein-deficient cells (Fig. 4e), while Viperin overexpression strongly induced the STAT1‒UBR5 interaction (Fig. 4e). Further studies showed that *UBR5* knockout attenuated STAT1 ubiquitination in Viperin protein-sufficient but not Viperin protein-deficient cells (Fig. 4f, g). In addition, we performed in vitro ubiquitination assays and the result confirmed that UBR5 acts as an E3 ubiquitin ligase for STAT1 (Fig. 4h). Furthermore, we analyzed the type of ubiquitin chain on STAT1 regulated by UBR5. The results showed that knockout of *UBR5* dramatically downregulated K48-linked polyubiquitination of STAT1 (Fig. 4i), which is consistent with the downregulation of STAT1 protein levels regulated by the E3 ligase UBR5. Importantly, Viperin-mediated downregulation of STAT1 protein levels was dependent on UBR5 (Fig. 4j). To demonstrate whether the blockade of UBR5 affects the levels of STAT1 and CVB3-induced SGK1 signaling, we employed *Ubr5*^−^^/−^ cells. The results showed that *UBR5* knockout abolished CVB3-induced STAT1 downregulation, and blocked CVB3-induced activation of the HF SGK1/KCNQ1 signaling (Fig. 4k, l). Taken all together, our findings suggest that CVB3-induced Viperin interacts with STAT1 and in turn promotes STAT1 ubiquitination and degradation dependently on UBR5.

### CVB3-encoded 3C cleaves UBE4A to rescue Viperin protein expression

**Fig. 5:**
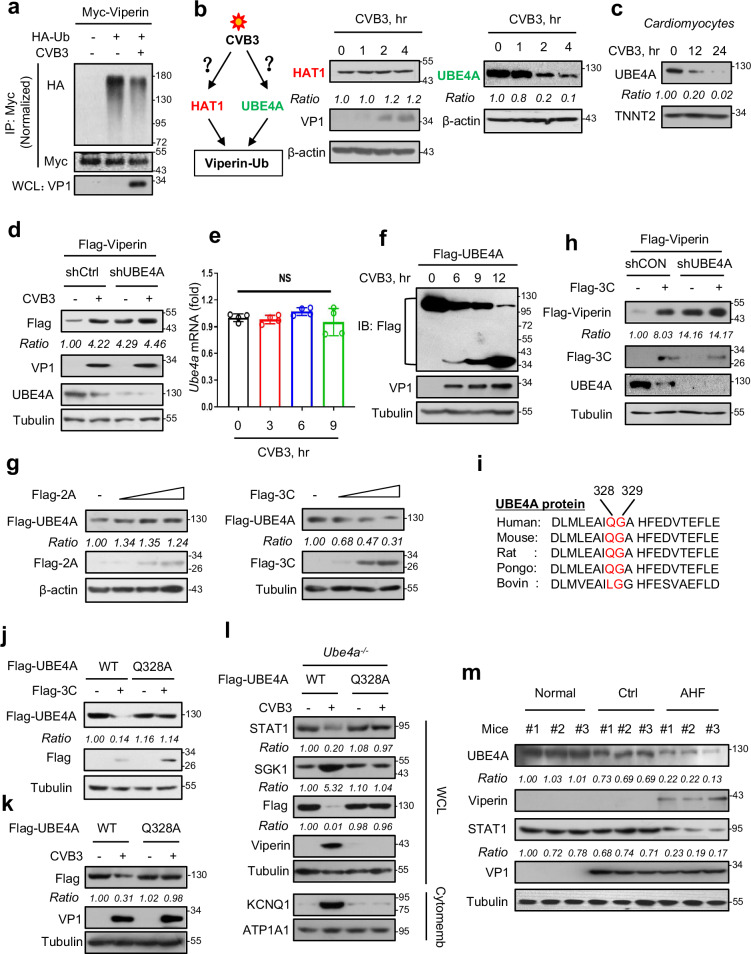
CVB3-encoded 3C lowers UBE4A levels to rescue Viperin protein expression. **a** Immunoprecipitation analysis of ubiquitination of Myc-Viperin in HEK293T cells transfected with Myc-Viperin and HA-Ub, followed by CVB3 infection (MOI = 10) for 12 h. **b** Western blot analysis of HAT1 and UBE4A levels in HL-1 cells infected with CVB3 (MOI = 10) as indicated. **c** Western blot analysis of UBE4A levels in mouse primary cardiomyocytes infected with CVB3 (MOI = 10) for 12 and 24 h. **d** Western blot analysis of Flag-Viperin levels in stable Flag-Viperin-expressing HL-1 cells transfected with shCtrl or shUBE4A and then infected with CVB3 (MOI = 10) for 12 h. **e** RT-qPCR analysis of *Ube4a* mRNA levels in HL-1 cells infected with CVB3 (MOI = 10) as indicated. **f** Western blot analysis of Flag-UBE4A and its cleaved fragment by Flag antibodies in HL-1 cells transfected with Flag-UBE4A, and then infected with CVB3 (MOI = 10) as indicated. **g** Western blot analysis of Flag-UBE4A levels in HL-1 cells transfected with Flag-UBE4A, together with Flag-tagged CVB3-encoded 2A (left) or 3C (right). **h** Western blot analysis of Flag-Viperin levels in HL-1 cells co-transfected with Flag-Viperin and Flag-3C, together with shCtrl or shUBE4A. **i** Multiple alignment of a putative motif targeted by CVB3 3C in different species of UBE4A. **j**, **k** Western blot analysis of Flag-UBE4A levels in HL-1 cells transfected with Flag-UBE4A (WT or Q328A), together with Flag-3C transfection (**j**) or CVB3 infection (MOI = 10, 12 h) (**k**). **l** Western blot analysis of STAT1, SGK1 and cytomembrane KCNQ1 levels in *Ube4a*^−/−^ cells transfected with Flag-UBE4A WT or Q328A, and then infected with or without CVB3 (MOI = 10) for 12 h. **m** Western blot analysis of UBE4A, Viperin and STAT1 levels in mice without CVB3 infection (Normal, *n* = 3), or in Ctrl mice (*n* = 3), or in AHF mice (*n* = 3). NS, not significant (*P* > 0.05), two-way analysis of variance (ANOVA). Data are shown as means ± SD of four biological replicates (**e**), or are representative of three independent experiments (**a**‒**d**, **f**‒**h**, **j**‒**m**).

An interesting question is how Viperin protein is restored in cardiomyocytes during CVB3 infection. Since it is well known that most of viruses can induce *Viperin* mRNA expression, we focused on Viperin protein induction specifically by CVB3. We first noticed that CVB3 infection attenuated Viperin protein ubiquitination (Fig. 5a). Given that Viperin ubiquitination has been reported to be tightly regulated by the HAT1-UBE4A axis^23^, we next asked whether CVB3 infection regulates HAT1 or UBE4A. We noticed that CVB3 infection partially reduced the levels of UBE4A but not HAT1 in cardiomyocytes in a time-dependent manner (Fig. 5b). Moreover, we confirmed that CVB3 infection decreased the protein level of UBE4A in primary cardiomyocytes in a time-dependent manner (Fig. 5c). Importantly, UBE4A deficiency restricted CVB3-induced upregulation of Flag-Viperin protein (Fig. 5d).

Further studies demonstrated that CVB3 infection did not significantly affect *Ube4a* mRNA levels in either cardiomyocytes or liver cells (Fig. 5e and Supplementary Fig. S5a). In addition, neither the proteasome inhibitor MG132 nor the lysosomal inhibitor Methylamine (MA) abolished CVB3-induced UBE4A reduction (Supplementary Fig. S5b, c). At the meanwhile, we noticed that CVB3 infection resulted in an increase in a small fragment of UBE4A protein (Fig. 5f), suggesting the possibility that CVB3 could cleave UBE4A protein. Thus, CVB3-encoded proteases, 2A and 3C, were further observed. The results showed that 3C but not 2A can downregulate UBE4A protein levels in a dose-dependent manner in both cardiomyocytes and liver cells (Fig. 5g and Supplementary Fig. S5d). Consistent with UBE4A downregulation, Viperin protein levels were upregulated by CVB3-encoded 3C (Fig. 5h), while UBE4A knockdown inhibited 3C-induced upregulation of Viperin protein (Fig. 5h), suggesting that 3C upregulates Viperin protein through UBE4A. To further confirm that 3C can cleave UBE4A protein, we next analyzed the amino acid sequence of UBE4A. A conserved QG sequence (Q_328_G_329_ in human UBE4A), which has been documented to be a potential target of 3C^33^, was observed (Fig. 5i). Thus, we generated the UBE4A-Q328A mutant and found that 3C lost the ability to downregulate the UBE4A-Q328A mutant (Fig. 5j). Consistently, CVB3 infection also cannot downregulate the levels of the UBE4A-Q328A mutant (Fig. 5k). In addition, CVB3 can downregulate STAT1 and upregulate SGK1/KCNQ1 signaling in *Ube4a*^−/−^ cells with UBE4A-WT transfection, but not in *Ube4a*^−/−^ cells transfected with UBE4A-Q328A (Fig. 5l). Moreover, we found that in UBE4A-WT cells, Viperin promoted HF SGK1 signaling during CVB3 infection, while *UBE4A* knockout abolished Viperin-mediated SGK1 activation (Supplementary Fig. S5e), suggesting a role of UBE4A in Viperin-mediated CVB3-induced HF.

Furthermore, we observed the in vivo effects of CVB3 infection on UBE4A, Viperin and STAT1 in hearts. Thus, we collected heart tissues from either three CVB3-infected mice with relatively normal LVFS levels or three AHF mice, which were from Fig. 1d. The results showed that as compared to the normal mice without CVB3 infection, CVB3 infection gradually lowered UBE4A protein levels (Fig. 5m). This was consistent with both the appearance of Viperin protein and a decrease in STAT1 protein levels in the AHF mice (Fig. 5m). Taken all together, these findings suggested that CVB3-encoded 3C lowers UBE4A levels in cells to rescue expression of Viperin protein, while the rescued Viperin protein can interact with STAT1, which in turn results in STAT1 degradation in cardiomyocytes.

### The interfering peptide (IP) VS-IP1 prevents CVB3-induced AHF in vivo

**Fig. 6:**
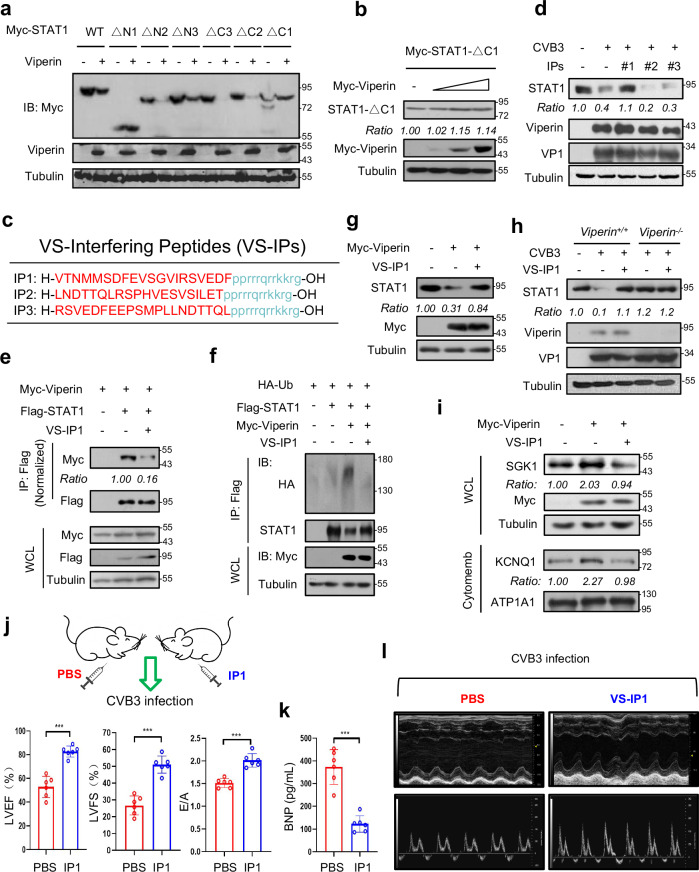
The IP VS-IP1 restricts CVB3-induced AHF in vivo. **a** Western blot analysis of Myc-STAT1 levels in HEK293T cells co-transfected with Viperin and Myc-STAT1 (WT or deletion mutants). **b** Western blot analysis of Myc-STAT1-ΔC1 levels in HL-1 cells co-transfected with Myc-STAT1-ΔC1 and increasing amounts of Myc-Viperin. **c** Design of the IPs to block Viperin‒STAT1 interaction. **d** Western blot analysis of STAT1, Viperin and UBE4A levels in HL-1 cells treated with IPs (200 μM) and CVB3 (MOI = 10) for 12 h. **e** Immunoprecipitation analysis of the interaction between Myc-Viperin and Flag-STAT1 in HL-1 cells transfected with Flag-STAT1 and Myc-Viperin and then treated with VS-IP1 (200 μM) for 12 h. **f** Immunoprecipitation analysis of STAT1 ubiquitination in HL-1 cells transfected with Flag-STAT1, Myc-Viperin and HA-Ub, and then treated with VS-IP1 (200 μM) for 12 h. **g** Western blot analysis of STAT1 levels in HL-1 cells transfected with Myc-Viperin and then treated with VS-IP1 (200 μM) for 12 h. **h** Western blot analysis of STAT1, Viperin and UBE4A in *Viperin*^*+/+*^ or *Viperin*^−/−^ HL-1 cells treated with VS-IP1 (200 μM) and CVB3 (MOI = 10) for 12 h. **i** Western blot analysis of SGK1 levels in HL-1 cells transfected with Myc-Viperin and then treated with VS-IP1 (200 μM) for 12 h. **j**‒**l** C57BL/6 mice were injected with PBS (*n* = 6, i.p.) or VS-IP1 (*n* = 6, i.p., 5 mg/kg) for 12 h and then infected with CVB3 (2 × 10^5^ PFU per gram body, i.p.) for 7 days. The LVEF, LVFS, and E/A ratio in mice were analyzed by echocardiography (**j**). Serum BNP concentrations were analyzed by ELISA (**k**). The representative images of the echocardiography were shown (**l**). ***P < 0.001, two-tailed unpaired Student’s *t*-test. Data are representative of three independent experiments (**a**, **b**, **d**‒**i**), or are shown as the means ± SEM for six individual mice (**j**, **k**).

We next sought to block the Viperin‒STAT1 interaction using IPs. To this end, we first determined the interaction domains that are essential for the Viperin‒STAT1 interaction. The results showed that deletion of the 4th part (4^#^) of the SAM domain of Viperin abolished STAT1 binding (Supplementary Fig. S6a), suggesting that STAT1 binds to the SAM-4^#^ domain of Viperin. Next, we generated a series of STAT1 deletion mutants (Supplementary Fig. S6b). We found that deletion of amino acids 704‒750 of STAT1 (STAT1-ΔC1) completely abolished Viperin binding (Supplementary Fig. S6b). Consistently, we found that Viperin overexpression cannot downregulate the levels of STAT1-ΔC1 (Fig. 6a, b), suggesting that the 704‒750 amino acid region of STAT1 is important for Viperin-mediated STAT1 reduction. Thus, we next designed three Viperin‒STAT1 IPs (VS-IPs), each of which covers different parts of the 704‒750 amino acid region of STAT1, and then we modified these VS-IPs to the D-retro-inverso (DRI) conformation (Fig. 6c). Peptides with the DRI conformation have been proven to be both well-tolerated and therapeutically effective in clinical trials^34^. We found that among these three IPs, VS-IP1 greatly blocked CVB3-induced downregulation of cellular STAT1 levels (Fig. 6d). Interaction analysis revealed that VS-IP1 inhibited the binding of Myc-Viperin with Flag-STAT1 (Fig. 6e and Supplementary Fig. S7a). Consistently, VS-IP1 lowered STAT1 ubiquitination induced by Viperin in not only cardiomyocytes but also HeLa cells (Fig. 6f and Supplementary Fig. S7b). As a consequence, VS-IP1 blocked Viperin-induced STAT1 protein reduction (Fig. 6g and Supplementary Fig. S7c). Importantly, during CVB3 infection, STAT1 protein levels were restored by VS-IP1 (Fig. 6h), while VS-IP1 had no effect on STAT1 protein levels in *Viperin*-knockout *Viperin*^−/^^−^ cells (Fig. 6h), suggesting that VS-IP1 regulates STAT1 levels dependently on Viperin. In addition, VS-IP1 inhibited Viperin-induced upregulation of the protein levels of both SGK1 and cytomembrane KCNQ1 in cardiomyocytes (Fig. 6i).

Next, C57BL/6 mice were i.p. injected with either the vehicle (PBS, *n* = 6) or VS-IP1 (*n* = 6). After 12 h, the mice were infected with CVB3 (2 × 10^5^ PFU per gram body mouse) for 7 days. The results showed that VS-IP1 treatment resulted in higher STAT1 protein levels and lower SGK1 protein levels in mouse heart tissues, compared with the PBS control group (Supplementary Fig. S7d). Importantly, some of the control mice with CVB3 infection have developed AHF, as shown by strong decreases in LVEF, LVFS and the E/A ratio (Fig. 6j), and a great increase in BNP levels (Fig. 6k), while VS-IP1 treatment maintained cardiac electrophysiology at relatively normal levels and therefore largely prevented cardiac dysfunction (Fig. 6j‒l). Collectively, these findings suggested that the IP VS-IP1 effectively restricts CVB3-induced AHF.

## Discussion

**Fig. 7:**
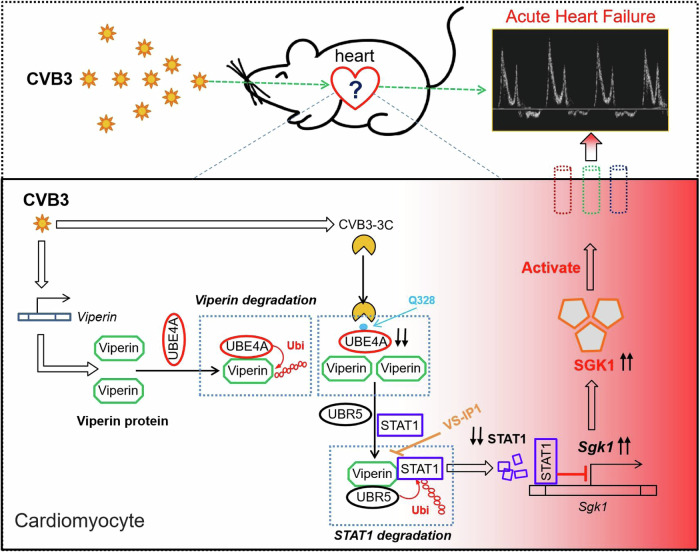
A proposed model. CVB3-encoded 3C protease cleaves UBE4A at Q328 residue to rescue Viperin, which in turn interacts with STAT1 and promotes STAT1 degradation by UBR5. STAT1 reduction in cardiomyocytes enhances SGK1 signaling and eventually promotes AHF. The IP VS-IP1 prevents CVB3-induced AHF by inhibiting Viperin-STAT1 interaction in vivo.

HF, including acute and CHF, seriously threatens people’s lives^1,2^. Animal models of CHF have been well established, which leads to great progress in elucidating the pathogenesis of CHF. AHF shows rapid onset and unremarkable changes in cardiac structure, thus making it difficult to study the regulatory mechanisms of AHF. CVB3 is a very common virus that causes AHF in clinic^15^. In this study, we revealed that Viperin protein is one of crucial regulators of AHF induced by CVB3 infection. Mechanistically, Viperin protein specifically induced by CVB3 activates SGK1-KCNQ1 signaling through STAT1 in cardiomyocytes, which leads to altered cardiac ion channels and eventually induces cardiac dysfunction and AHF (Fig. 7).

Although the mechanisms of HF need to be further explored, it is currently clear that electrical and mechanical cardiac abnormalities are necessary for the occurrence of AHF^18,21,22^. However, the critical molecular events that mediate CVB3-induced electrical and mechanical cardiac abnormalities remain largely unknown. Interestingly, this study uncovered that an important antiviral protein, Viperin, can regulate SGK1 that has been demonstrated to be both necessary and sufficient to induce the adverse electrical and mechanical remodeling observed in HF. Viperin has been demonstrated to be a powerful antiviral protein against a broad spectrum of viruses^35–37^. Thus, this study revealed a previously unknown function of Viperin, that is, regulating myocardial electrical/mechanical abnormalities and AHF.

This study shows an intriguing switch from Viperin‒UBE4 to Viperin‒STAT1‒UBR5 interaction during CVB3 infection. This intracellular switch results in STAT1 degradation and in turn activates SGK1 signaling. SGK1 has been demonstrated to be necessary and sufficient to induce hallmarks of electric and mechanical remodeling in HF^29^. As a consequence, CVB3-induced Viperin protein mediates AHF. In addition, it is possible that other CVB3-induced factors could also contribute to the final occurrence of AHF. Thus, we speculated that continuous Viperin accumulation and SGK1 activation in cardiomyocytes, together with other CVB3-induced factors, eventually lead to AHF.

Due to the insufficient understanding of the mechanisms of AHF, there are still no specific strategies to treat AHF. In this study, we designed an IP, VS-IP1, which is able to restrict CVB3-induced cardiac dysfunction and AHF. To our knowledge, this is the first IP showing the potential to treat AHF. In summary, this study reveals the crucial role of Viperin in regulating cardiac dysfunction and AHF through SGK1, and designed an IP VS-IP1 that prevents CVB3-induced AHF.

## Materials and methods

### Human samples

This study included the serum samples from 34 CVB3 positive patients without AHF (16–36 years old, mean = 26; 15 females and 19 males) and 20 CVB3 positive patients with AHF (18–36 years old, mean = 27; 13 females and 7 males) from the department of Cardiology in the Zhongshan Hospital of Fudan University in Shanghai, China. The study was approved by the Research Ethics Committee of the Zhongshan Hospital of Fudan University in Shanghai. All diagnoses about CVB3 infection and AHF were performed at the department of Cardiology in the Zhongshan Hospital of Fudan University in Shanghai, China.

### Mice

*Viperin*^−/−^ mice in a C57BL/6 background were generated by the Cyagen Biosciences Inc. (Guangzhou, China). Wild-type C57BL/6 mice were purchased from the Laboratory Animal Center of Soochow University (the ethics approval number: 202305A0657, 202307A0236). Mice were maintained and bred in special-pathogen-free conditions in the Experimental Animal Center of Soochow University. 6‒8 months old mice were used for most experiments. Animal care and use protocols are adhered to the National Regulations for the Administration of Affairs Concerning Experimental Animals. All protocols and procedures for mouse studies were performed in accordance with the Laboratory Animal Management Regulations with approval of the Scientific Investigation Board of Soochow University.

### Primary cells from mice

For the mouse embryonic fibroblasts (MEFs), *Viperin*^−/−^ embryos were obtained from the pregnant 13-day mice. Briefly, the embryos were cut into pieces and digested in 0.05% trypsin/EDTA with 100 units of DNase I, and then cultured in DMEM medium containing 10% FBS, 200 mM L-glutamine, 100 units/ml penicillin and 100 µg/mL streptomycin. The primary cells were isolated from mouse tissues, which were cut into small pieces and digested by the erythrocyte lysis buffers. After centrifugation, the cells were collected and then cultured in RPMI medium until further experiments.

### Cell culture and maintenance, transfection

HEK293T, HepG2 and HeLa cells were obtained from the American Type Culture Collection. HL-1 cells were gifts from Dr. Sidong Xiong (Soochow University). The cells were cultured in Dulbecco’s modified Eagle’s medium (DMEM; HyClone) supplemented with 10% FBS (GIBCO, Life Technologies), 100 units/mL penicillin and 100 µg/mL streptomycin. THP1 cells were obtained from ATCC and were cultured in RPMI 1640 medium (HyClone). All cells were cultured at 37 °C under 5% CO_2_. All transient transfections were carried out using either Lipofectamine 2000 (Thermo Fisher), or LongTrans (Ucallm), or PEI (Polyetherimide) according to manufacturer’s instructions.

### Expression constructs and reagents

HA-tagged mouse Viperin and HA-tagged mouse Viperin-Δ(1-42) were nice gifts from Dr. Peter Cresswell (Yale University). Myc-tagged mouse Viperin was a nice gift from Dr. Zhenghong Yuan (Fudan University, China). Flag-tagged human Viperin was a nice gift from Dr. Chunfu Zheng (Fujian Medical University, China). Myc-tagged human Viperin (WT and the deletion mutants) and Flag-HA (FH)-tagged Viperin were generated using PCR amplification from Flag-tagged human Viperin. Flag-STAT1 and Myc-His-tagged human STAT1 deletion mutants were generated using PCR amplification from Myc-His-tagged STAT1-WT. Flag-UBE4A was kindly provided by Dr. Wei Li (Institute of Zoology, Chinese Academy of Sciences). HA-ubiquitin (HA-Ub) was a nice gift from Dr. Lingqiang Zhang (State Key Laboratory of Proteomics, Beijing). Flag-2A (CVB3) and Flag-3C (CVB3) were gifts from Dr. Sidong Xiong (Institutes of Biology and Medical Sciences, Soochow University, China). All shRNAs against Viperin (shViperin) or STAT1 (shSTAT1) were purchased from GENECHEM (Shanghai, China): shViperin 1# sequence: TAGCTACCAAGAGGAGAAA; shViperin 2# sequence: GCTTTCTGAACTGTAGAAA; shViperin 3# sequence: CTGAATCTAACCAGAAGAT; shSTAT1 sequence: ACAGAAATACACCTACGAA. All mutations were generated by the QuickChange Lightning site-Directed Mutagenesis Kit (Stratagene, 210518). All plasmids were confirmed by DNA sequencing. CHX, MG132, puromycin, Polybrene and other chemicals were purchased from Sigma.

### Echocardiography

Mice were lightly anesthetized with inhalant isoflurane (0.5%–1%). Then, mice were imaged using a 40-MHz linear array transducer attached to a preclinical ultrasound system (Vevo 2100, FUJIFILM VisualSonics, Toronto, Canada) with nominal in-plane spatial resolution of 40 mm (axial) × 380 mm (lateral). Ejection fraction (EF) and fractional shortening (FS) were analyzed. The pulsed wave Doppler measurements of maximal early (E) and late (A) transmitral velocities in diastole were carried out in the apical view with the cursor at mitral valve inflow. Finally, mice were euthanized by cervical dislocation under anesthesia with inhaled isofurane (3%–5%) for futher studies.

### Cardiomyocyte-specific gene expression by AAV9

In vivo gene overexpression was achieved by the AAV9 vectors. Recombinant adeno-associated virus serotype 9 (AAV9) vectors carrying GFP or Viperin with a cardiac-specific promoter c-TNTp (AAV9-cTNTp-Ctrl, AAV9-cTNTp-Viperin) were manufactured by the Genechem Co. Ltd (Shanghai, China). AAV9-cTNTp-Ctrl served as a negative control, and AAV9-cTNTp-Viperin vectors (6 × 10^11^ vector genomes (vg)/mouse) were delivered by intravenously injection via tail vein. After 14 days, the mice were anesthetized with inhaled isoflurane (1%) and imaged by echocardiography. After that, the hearts were harvested for further analysis.

### Mass spectrometry

For identification of Viperin-binding proteins, HEK293T cells were transfected with empty vectors or Flag-Viperin. After 48 h, cells were harvested by Nonidet P-40 lysis buffer containing 150 mM NaCl, 20 mM Tris-HCl (pH7.4), 1% NP-40, 0.5 mM EDTA, PMSF (50 µg/mL) and protease inhibitors mixtures (Sigma-Aldrich). Next, the M2 affinity gel (Sigma-Aldrich, A2220) was used to pull down Flag-Viperin from the whole cell lysates. SDS-PAGE gels were stained with the Silver Staining kits (Beyotime, P0017S). The gel bands from control and experimental samples were carefully excised, and then were digested with trypsin. The resulting tryptic peptides were purified using C18 Zip-Tip and analyzed by an Orbitrap Elite hybrid mass spectrometer (Thermo Fisher) coupled with a Dionex LC.

### Quantitative proteomic analysis

Protein-peptide identification and quantification were performed by the PTM BioLab Inc. (Hangzhou, China). Sample was sonicated three times on ice using a high intensity ultrasonic processor (Scientz) in lysis buffer (8 M urea, 1% Protease Inhibitor Cocktail). The remaining debris was removed by centrifugation at 12,000× *g* at 4 °C for 10 min. Finally, the supernatant was collected and the protein concentration was determined with a BCA kit. For digestion, the protein solution was reduced with 5 mM dithiothreitol for 30 min at 56 °C and alkylated with 11 mM iodoacetamide for 15 min at room temperature in the dark. After trypsin digestion, peptide was desalted by Strata X C18 SPE column (Phenomenex) and vacuum-dried. Peptide was reconstituted in 0.5 M TEAB. The peptide mixtures were then incubated for 2 h at room temperature and pooled, desalted and dried by vacuum centrifugation. The sample was fractionated into fractions by high pH reverse-phase HPLC using Agilent 300 Extend C18 column (5 µm particles, 4.6 mm ID, 250 mm length). Peptides were separated with a gradient of 8% to 32% acetonitrile in 10 mM ammonium bicarbonate pH 9 over 60 min into 60 fractions. Then, the peptides were combined into 12 fractions and dried by vacuum centrifuging.

The tryptic peptides were dissolved in 0.1% formic acid and 2% acetonitrile (solvent A), directly loaded onto a home-made reversed-phase analytical column (15-cm length, 75 µm, inner diameter). The peptides were subjected to NSI source followed by tandem mass spectrometry (MS/MS) in Orbitrap Exploris™ 480 coupled online to the UPLC. Peptides were then selected for MS/MS using NCE setting as 28 and the fragments were detected in the Orbitrap at a resolution of 15,000. A data-dependent procedure that alternated between one MS scan was followed by 25 MS/MS scans with 15.0 s dynamic exclusion. Automatic gain control was set at 5E4. Fixed first mass was set as 100 m/z. The resulting MS/MS data were processed using Proteome Discoverer (v2.4.1.15). Tandem mass spectra were searched against the mouse uniprot database (Mus_musculus_10090_SP_20210721.fasta) concatenated with reverse decoy database.

### CRISPR-Cas9-mediated genome editing

The lentiCRISPRv2 vector was a nice gift from Dr. Fangfang Zhou (Soochow University, China). Small guide RNAs targeting *Viperin*, *Ube4a*, *Sgk1*, *Cnot1*, *Stat1*, *Atm*, *Stat3*, and *Tcf25* gene targets were cloned into the lentiCRISPRv2 vector and then transfected into HEK293T cells. Forty-eight hours after transfection, the supernatant containing lentiviruses was collected. Furthermore, HL-1 or HEK293T cells were infected by the supernatant. After 48 h, these cells were split into 96-well plates and were cultured under puromycin selection until further experiments.

The gRNA sequences are as follows: *Viperin*: ATTGCTCACGAT GCTCACGC; *SGK1*: GCTACATGCCTCTGATAAGC; *CNOT1*: GAATCTTGAC TCGCTCTCGC; *STAT1*: TCCCATTACAGGCTCAGTCG; *ATM*: CCAAGGCTA TTCAGTGTGCG; *STAT3*: AGATTGCCCGGATTGTGGCC; *TCF25*: ATTACCG CAGACCCGAGAAC; *UBE4A*: CCGCTCATTCCGATCACAGC; *HUWE1*: GACCTGTTGGACCGCTTCGA; *UBR5*: CCACCCCCCTTTGAATGTAT; *RNF213*: ACTCACTTCTTGGACGGTCC; *LTN1*: TGTAGATTCTGATTTCC GAA; *UBE3C*: CGACTTCAAGACGCGGCCCA; *PJA2*: TGTTCACTACAAA TATGCCC; *RNF123*: CATTGTCCACCTGTAGCAAC; *HECTD1*: TATCTGCGG AATGTACCCGA; *RNF187*: CGTCCGGTCGCCGGCCGTCT.

### Viral infection in vitro

CVB3 (Nancy strain) was from Dr. Sidong Xiong (Soochow University, China). VSV and SeV were gifts from Dr. Chen Wang (China Pharmaceutical University). Influenza A virus (H1N1, PR/8/34) was a gift from Dr. Jianfeng Dai (Soochow University, China). HSV-1 was obtained from Chunfu Zheng (Fujian Medical University). After washing twice by 1× PBS, cells were cultured in serum-free medium and then infected with different viruses diluted with DMEM serially for 1.5 h. Then the supernatant was removed and the cells were further cultured in DMEM medium with 10% FBS until harvest.

### Viral infection in vivo

C57BL/6 mice were injected i.p. with CVB3 (2 × 10^5^ PFU per gram body mouse). Then the mice were anesthetized with inhaled isoflurane (1%) and imaged by echocardiography. After that, the hearts were harvested for further analysis.

### CHX pulse chase assay

The half-life of STAT1 protein was determined by a CHX pulse chase assay. Briefly, cells were transfected with Flag-STAT1 and HA-Viperin plasmids. After 48 h, cells were treated with DMSO or CHX (50 µg/mL) for 0, 12, and 36 h. Then cells were harvested and the boiled whole cell lysates were analyzed by western blot assay.

### In vivo ubiquitination assay

Cells were transfected with the corresponding plasmids, together with or without HA-ubiquitin (Ub). Forty-eight or seventy-two hours after transfection, cells were harvested in RIPA strong lysis buffer with Nethylmaleimide (10 mM), PMSF (50 µg/mL), and protease inhibitor mixtures (Sigma-Aldrich). For STAT1 ubiquitination analysis, immunoprecipitation was performed by a specific antibody (Ab) on a rotor at 4 °C. After washing three times with high-salt (500 mM NaCl) washing buffer and subsequent twice with the normal salt (150 mM NaCl) washing buffer, the immunoprecipitates were analyzed by western blot assay using the anti-HA or anti-Ub Ab.

### Cytomembrane protein extraction

The membrane protein of cells was extracted by a Membrane and Cytosol Protein Extraction Kit (Beyotime, #20127ES50, China) following the manufacturer’s protocol. Then the protein concentrations were measured by a BCA Protein Assay Kit.

### RNA extraction and RT-qPCR

RNAs were extracted from different cells or mouse tissues using TRIzol reagent (Invitrogen). The cDNA was produced by reverse transcription using oligo (dT) or random primer according to the manufacturer’s instructions (Invitrogen) using 1 µg of total RNAs.

RT-qPCR was conducted with the SYBR Green (Selleck) using a StepOne Plus real-time PCR system (Applied Bioscience). The relative gene expression levels were calculated using change-in-cycling-threshold (2^−^^ΔΔCt^) method. Quantification of all target genes was normalized to the control gene β-actin, and all data are shown as fold change normalized to that in either unstimulated or uninfected cells accordingly. The results were analyzed from three independent experiments and are shown as the average mean ± SD. The primer sequences are as following:

Human *Ube4a:*

Forward: 5′-GCATCCCTAGCCGTTGTGTGT-3′

Reverse: 5′-TGTGCCTCTCTCCTGCATCTCG-3′

Mouse *Viperin*:

Forward: 5′-CAGGCTGGTTTGGAGA AGATCAAC-3′

Reverse: 5′-TACTCCCCATAGTCCTTG AACCATC-3′

Human *Sgk1*:

Forward: 5′-GCTGAAATAGCCAGTGCCTTGG-3′

Reverse: 5′-GTTCTCCTTGCAGAGTCCGAAG-3′

Mouse *Sgk1*:

Forward: 5′-CTCATTCCAGACCGCTGACAAAC-3′

Reverse: 5′-CCAAGGCACTGGCTATTTCAGC-3′

*β-actin*:

Forward: 5′-ACCAACTGGGACGACATGGAGAAA-3′

Reverse: 5′-ATAGCACAGCCTGGATAGCAACG-3′

### Western blot assay and ELISA

Equal amounts of total proteins were subjected to SDS-PAGE and then transferred to PVDF membranes (Millipore). Membranes were blocked with either 5% skim milk or 5% BSA for 0.5 h at room temperature, and probed with the corresponding primary antibodies, followed by the respective HRP-conjugated goat anti-mouse or anti-rabbit (Bioworld) secondary antibody. After washing three times with PBST, the membrane was visualized with the ECL Prime (Thermo Scientific). The antibodies with indicated dilutions were as follows: anti-Viperin (Abcam, ab107359, 1:1000), anti-SGK1 (Abcam, ab59337, 1:1000), anti-KCNQ1 (Affinity, DF7568, 1:1000), anti-ATP1A1 (Affinity, AF6109, 1:1000), anti-Flag (Sigma, F7425, 1:5000), anti-STAT1 (Cell Signaling Technology, 8826, 1:5000), anti-HA (Sigma, ab9110, 1:5000), anti-Myc (Abmart, M20002, 1:5000), Ubiquitin (Ub) (Santa Cruz, sc-8017, 1:500), anti-UBE4A (Santa Cruz, sc-365904, 1:1000), anti-HAT1 (Santa Cruz, sc-376268, 1:1000), anti-UBR5 (Affinity, AF0268, 1:1000), anti-β-Actin (Proteintech, 66009, 1:5000) and anti-Tubulin (Proteintech, 66031-1-Ig, 1:5000). BNP ELISA kit (Elabscience, E-EL-M0204c) was from the Elabscience. Human Viperin ELISA kit (FineTest, EH2283) was from the FineTest.

### Immunoprecipitation

Cells were harvested using the lysis buffer containing 150 mM NaCl, 20 mM Tris-HCl (pH 7.4), 1% Nonidet P-40, 0.5 mM EDTA, PMSF (50 µg/mL) and protease inhibitors mixtures (Sigma). When protein ubiquitination was examined, RIPA buffer containing SDS (Beyotime) was used and then N-ethylmaleimide (10 mM) was added into the lysis buffer. The cell lysates were incubated with specific Abs on a rotor at 4 °C. Protein G-agarose beads (Millipore; 16–266) were firstly washed twice and then were added into the supernatant. The mixture was incubated for 2–3 h on a rotor at 4 °C. For immunoprecipitation of Flag/Myc/HA-tagged proteins, either M2 affinity gel (A2220; Sigma-Aldrich), or Myc/HA magnetic beads (Selleck) were added to the cell lysates. After washing three times with the washing buffer containing 150 mM NaCl, the immunoprecipitates were analyzed by western blot assay. For immunoprecipitation normalization: target proteins were firstly immunoprecipitated and then were serially diluted with loading buffer for analysis of target protein levels by immunoblotting. According to the immunoblotting results, the same amount of immunoprecipitated target proteins are loaded for analysis of interaction or ubiquitination. The whole cell lysates (30 μg) are used for an input control.

### IPs

The VS-IPs are as follows:

VS-IP1: H-VTNMMSDFEVSGVIRSVEDFpprrrqrrkkrg-OH;

VS-IP2: H-LNDTTQLRSPHVESVSILETpprrrqrrkkrg-OH;

VS-IP3: H-RSVEDFEEPSMPLLNDTTQLpprrrqrrkkrg-OH;

VS-IPs were manufactured by GL Biochem (Shanghai, China) at > 95% purity and stored at −20 °C in 1 mg powder aliquots to avoid freeze-thawing artifacts. For in vitro experiments, IPs were dissolved in PBS to generate a 25 mM stock. For in vivo experiments, IPs were dissolved in PBS to generate a 5 mg/mL stock solution, which were kept on ice until injection. Before injection, the solution was brought to room temperature.

### Construction of stable cell lines

HEK293T cells were transfected with pOZ-FH-C-puro (empty vectors; Addgene) or Flag-HA-Viperin, together with PCL Ampho plasmids. After 36 h, the supernatant was collected and used to infect either HeLa or HEK293T cells. The infected cells were cultured for 24 h with polybrene (8 µg/mL). After removing the culture medium, the cells were cultured in 10% FBS without polybrene for 24 h. Then cells were cultured in 10% FBS with puromycin (1.5 µg/mL) for two weeks’ selection. The stable cell lines were maintained in 10% FBS medium with puromycin (1.5 µg/mL) until 2 days before further experiments.

### In vitro ubiquitination assay

Cells were transfected with Flag-STAT1. Forty-eight hours after transfection, cells were harvested in lysis buffer with PMSF. To analyze the effect of UBR5 on the ubiquitination of STAT1, Flag-STAT1 proteins were immunoprecipitated by Flag M2 beads. After washing three times with high-salt washing buffer (500 μM of NaCl), Flag-STAT1 immunoprecipitates were eluted by Flag peptides (Sigma, Cat# F3290). The retrieved proteins were added into each reaction buffer containing 100 nM E1 ligase (UBE1, Boston Biochem, E306), 0.5 mM E2 ligase (UbcH5a, Boston Biochem, E2-615), 1× Ubiquitin Conjugation Reaction Buffer containing ATP (Boston Biochem, SK-10) and UBR5 (Cell Signaling Technology, Cat# 65344). Reactions were allowed to proceed at 37 °C for 1 h and were stopped by boiling for 5 min. Flag-STAT1 proteins were immunoprecipitated at 4 °C for further western blot analysis.

### GST pull-down assay

HL-1 cells were infected with CVB3 (MOI = 10) for 24 h and cells were harvested in lysis buffer to get whole cell lysates (WCL). GST-STAT1 recombinant proteins (MCE, HY-P73628) or GST recombinant proteins (MCE, HY-P70270) were added to the above WCL. Then GST beads were washed three times by washing buffer and were added to the WCL. After flipping overnight in the shaker at 4°, the supernatant was removed and the beads were washed three times with washing buffer. Viperin proteins were analyzed by immunoblotting using an anti-Viperin antibody.

### siRNA injection in mice via the tail vein

siCtrl (sense 5′-3′: UUCUCCGAACGUGUCACGUdTdT, antisense 5′-3′: ACGUGACACGUUCGGAGAAdTdT) and siSTAT1 (sense 5′-3′: GCCGAGAA CAUACCAGAGAAUTT, antisense 5′-3′: AUUCUCUGGUAUGUUCUCGGCTT) were synthesized in Chemshine (Sichuan, China). The siCtrl and siSTAT1 were injected into mice via the tail vein at a dose of 0.1 mg per mouse once a day for four consecutive days.

### Mouse primary cardiomyocytes

Mouse hearts were removed and submerged in 60 mm dish of PBS buffer. Rinse thoroughly with PBS to remove blood. The tissue was cut into 1 mm^3^ pieces gently. Five milliliters of enzyme (0.1% trypsin and 0.1% collagenase II) was added to the pieces and digested for 8 min in 37 °C incubator. The supernatant was collected, and digestion was terminated using 5 mL of 10% FBS medium. In the remaining tissue block, 5 mL of new digestive enzymes were added to continue digestion, as above, until digestion was complete. All supernatants were collected and filtered using a 74 μm nylon mesh. Cells were resuspended by adding 10 mL of complete medium and transferred to sterile Petri dishes. The cells were cultured at 5% CO_2_, 37 °C for 90 min to remove fibroblast contamination. Cardiomyocytes were cultured in medium containing 10% fetal bovine serum at 37 °C.

### Statistical analysis

Comparison between different groups was analyzed using the two-tailed unpaired Student’s *t*-test. A one-way analysis of variance (ANOVA) was used for comparisons between multiple groups in a single factor, and two-way analysis of variance was used for half-life experiments. Values of *P* < 0.05 were considered statistically significant. *P < 0.05, **P < 0.01, ***P < 0.001; NS, not significant.

## Supplementary information

Supplementary Information

## Data Availability

The data for quantitative proteomic analysis generated in this study have been deposited to the ProteomeXchange Consortium via the PRIDE partner repository with the dataset identifier PXD036898 (http://www.ebi.ac.uk/pride/archive/projects/PXD036898). All data generated or analyzed during this study are included in Figs. 1–7 and Supplementary Figs. S1–S7. Additional datasets that support the findings of this study are available from the corresponding author upon reasonable request.
