# Supplementary material for: Targeting Viperin prevents coxsackievirus B3-induced acute heart failure
Source: Cell Discov. 2025 Apr 8;11:34. doi: 10.1038/s41421-025-00778-0 (PMC11977219; doi:10.1038/s41421-025-00778-0)
Supplement: Supplementary file 1 — Supplementary Information [file 41421_2025_778_MOESM1_ESM.pdf]

Supplementary Fig. S1

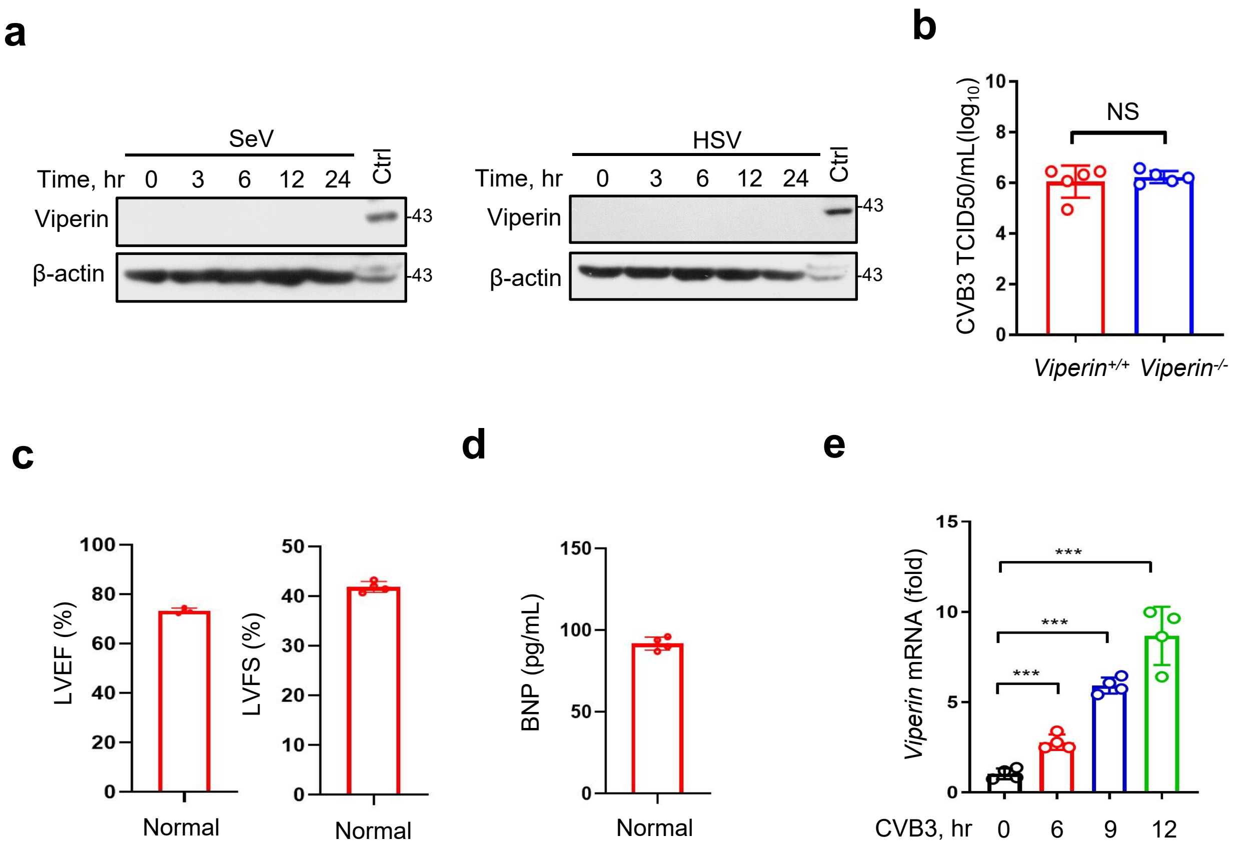

Supplementary Fig. S1. CVB3 induces Viperin expression.

a Western blot analysis of Viperin (Viperin) protein expression in HL-1 cells infected with SeV (MOI = 1.0) or HSV-1 (MOI = 1.0) for different times. The controls (Ctrl) are whole cell lysates from MEF cells treated with IFNβ.

b *Viperin*$^{+/+}$ (n = 5) or *Viperin*$^{-/-}$ (n = 5) mice were intraperitoneally infected with CVB3 ($2x10^5$ PFU per gram body, i.p.) for 3 days. Virus titers in the blood were analyzed by the TCID50 assay.

c,d The normal LVEF and LVFS levels in mice (n = 4) without CVB3 infection were analyzed by echocardiography (**c**) and serum BNP concentrations were analyzed by the ELISA kit (**d**).

e RT-qPCR analysis of Viperin mRNA levels in mouse primary cardiomyocytes infected with CVB3 (MOI = 10) as indicated.

NS, not significant ($p > 0.05$), ***$p < 0.001$ (two-tailed unpaired Student's *t*-test). The graphs show means ± SEM for four or five individual mice (**b**-**d**). Data are shown as means ± SD of four biological replicates (**e**), or are representative of three independent experiments (**a**).

Supplementary Fig. S2

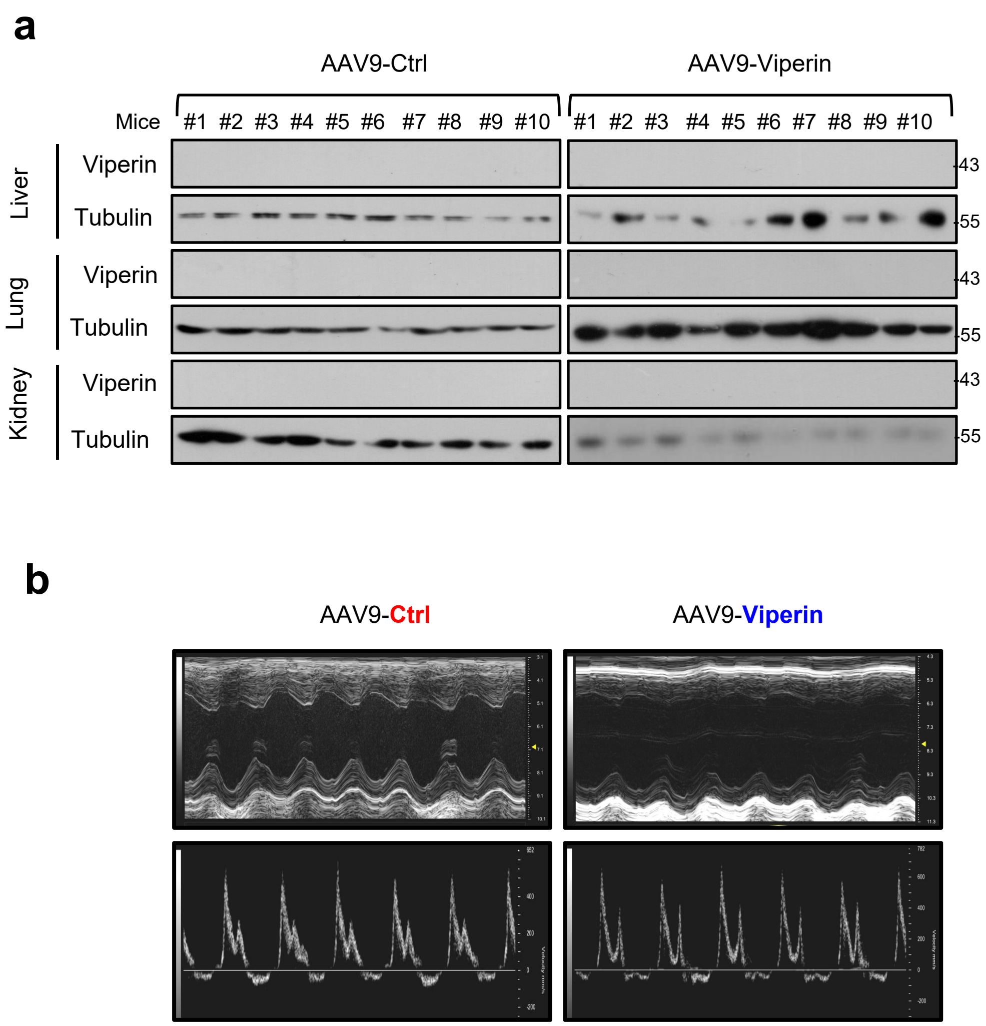

Supplementary Fig. S2. Cardiac-specific overexpression of Viperin mediates cardiac dysfunction.

a AAV9-Viperin with cTNTp (AAV9-Viperin) or the empty vector (AAV9-Ctrl) was injected into the mice (n = 10). After 14 days, Viperin protein levels in mouse liver, lung and kidney tissues were analyzed by western blot.

b AAV9-Ctrl and AAV9-Viperin with the promoter cTNTp was injected into the mice. After 14 days, the representative images of the echocardiography was analyzed in AAV9-Ctrl mice and AAV9-Viperin mice.

Data are representative of three independent experiments (**a**, **b**).

Supplementary Fig. S3

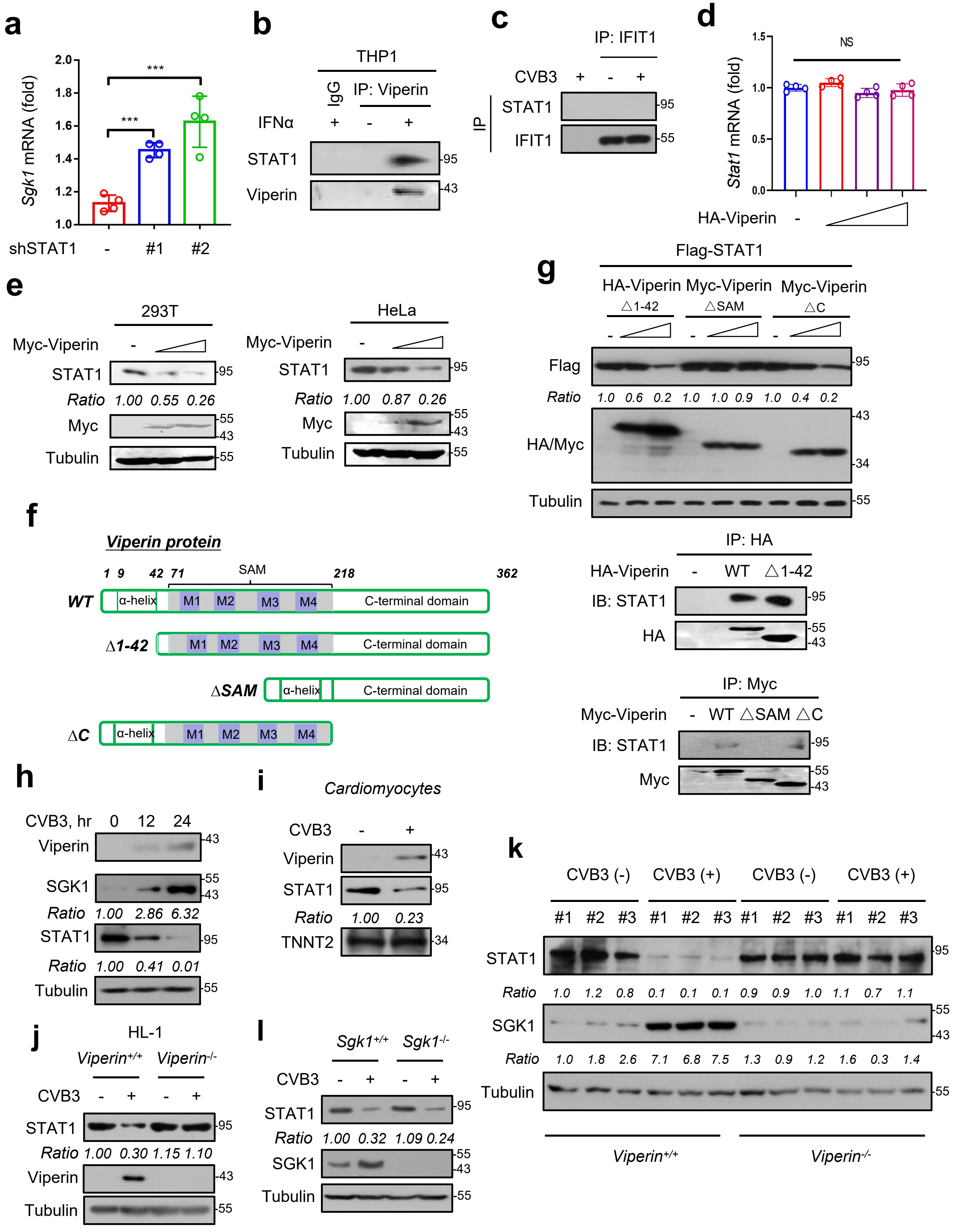

Supplementary Fig. S3. Viperin interacts with and downregulates STAT1.

a RT-qPCR analysis of *Sgk1* mRNA levels in HEK293T cells transfected with shCtrl (-) or shSTAT1 (#1 and #2) as indicated.

b Immunoprecipitation (IP) analysis of the interaction between Viperin and STAT1 in THP1 cells treated with IFNα (1,000 IU/ml) as indicated.

c IP analysis of the interaction between STAT1 and IFIT1 in HL-1 cells infected with CVB3 (MOI = 10) as indicated.

d RT-qPCR analysis of STAT1 mRNA in HL-1 cells transfected with increasing amounts of HA-Viperin.

e Western blot analysis of STAT1 levels in HEK293T (left) and HeLa (right) cells transfected with increasing amount of Myc-Viperin.

f Overview of Viperin-wild type (WT) and Viperin deletion mutants (left). HEK293T cells were transfected with the vector (-) or HA-Viperin (WT or its deletion mutants). IP was used to analyze the interaction between STAT1 and HA-Viperin (right).

g Western blot analysis of Flag-STAT1 levels in HEK293T cells co-transfected with Flag-STAT1 and different Myc-Viperin deletion mutants.

h Western blot analysis of Viperin, SGK1 and STAT1 levels in HL-1 cells infected with CVB3 (MOI = 10) for 12 and 24 h.

i Western blot analysis of Viperin and STAT1 levels in primary cardiomyocytes infected with CVB3 (MOI = 10) for 12 h.

j Western blot analysis of STAT1 and Viperin levels in *Viperin*$^{+/+}$ or *Viperin*$^{-/-}$ HL-1 cells infected with or without CVB3 (MOI = 10) for 12 h.

k, l Western blot analysis of STAT1 and SGK1 in *Viperin*$^{+/+}$ or *Viperin*$^{-/-}$ mice (**k**) and in *Sgk1*$^{+/+}$ or *Sgk1*$^{-/-}$ HL-1 cells (**l**) infected with or without CVB3 (MOI = 10) for 12 h . NS, not significant (p > 0.05), ***p < 0.001, two-tailed unpaired Student's *t*-test (**a**), one-way analysis of variance (ANOVA) (**d**). Data are shown as means ± SD of four biological replicates (**a**, **d**), or are representative of three independent experiments (**b**, **c**, **e-l**).

Supplementary Fig. S4

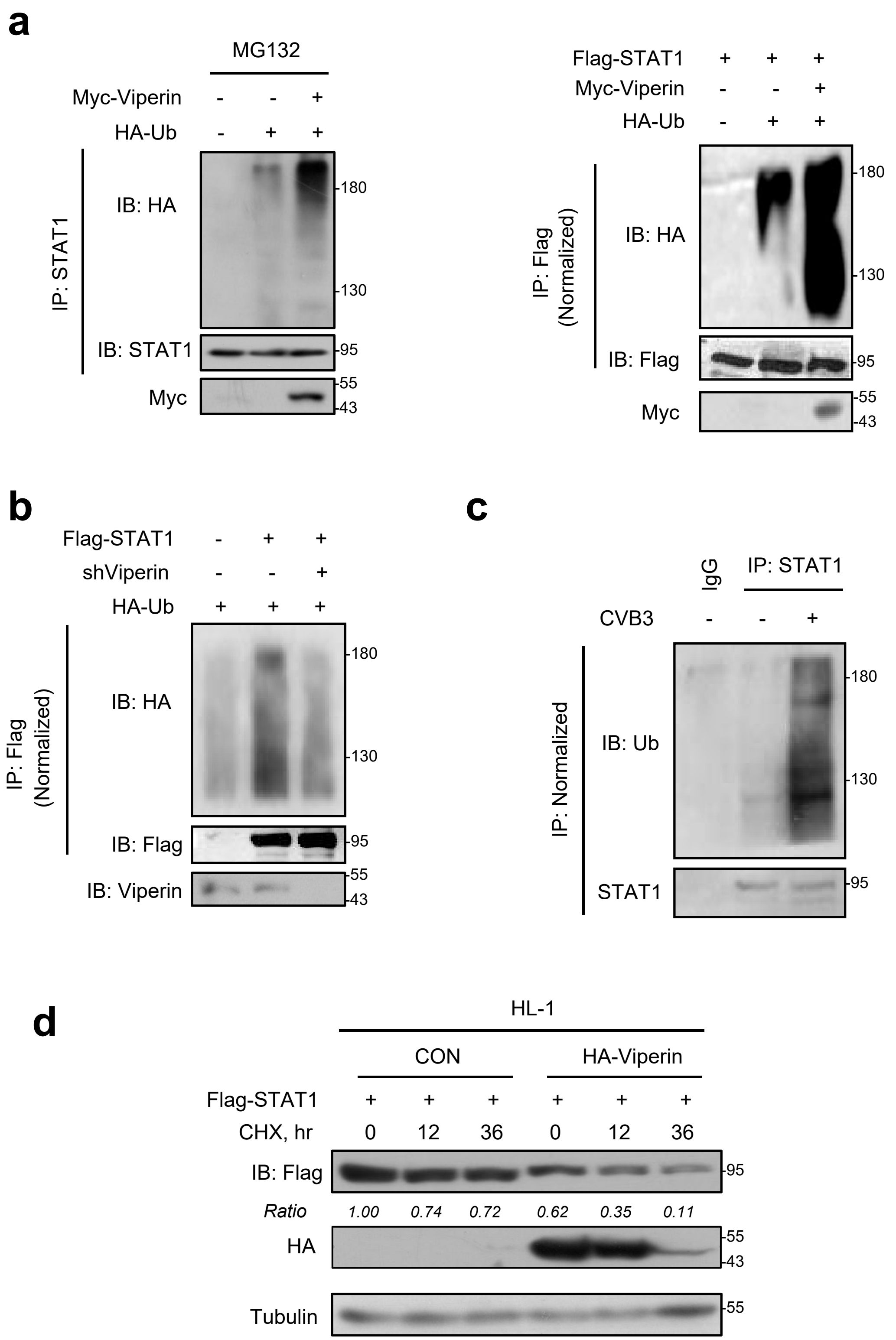

Supplementary Fig. S4. Viperin promotes STAT1 ubiquitination and lowers STAT1 protein stability.

a IP analysis of STAT1 ubiquitination in HEK293T cells co-transfected with Myc-Viperin and HA-Ub and then treated with MG132 (left), or co-transfected with Flag-STAT1, Myc-Viperin and HA-Ub (right).

b IP analysis of STAT1 ubiquitination in 2fTGH cells co-transfected with Flag-STAT1 and HA-Ub, together with shViperin.

c IP analysis of STAT1 ubiquitination in HepG2 cells infected with CVB3 (MOI = 10) for 12 h.

d Western blot analysis of STAT1 levels in HL-1 cells transfected with Flag-STAT1 and HA-Viperin, followed by CHX treatment (50 μM) for 12 and 36 h.

Data are representative of three independent experiments (**a**-**d**).

Supplementary Fig. S5

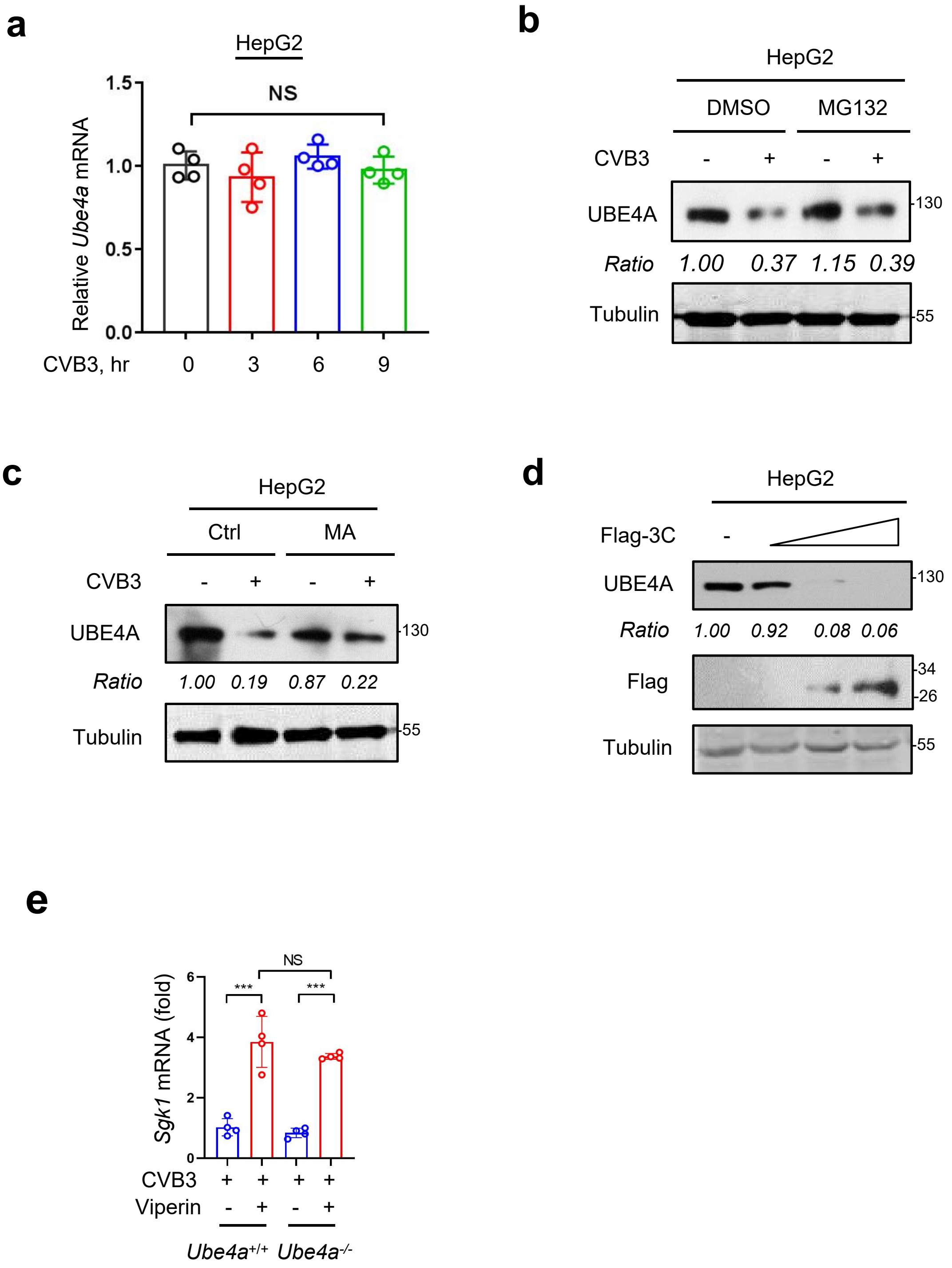

Supplementary Fig. S5. CVB3-encoded 3C reduces UBE4A levels.

a RT-qPCR analysis of *Ube4a* mRNA levels in HepG2 cells infected with CVB3 (MOI = 10) as indicated.

b Western blot analysis of UBE4A levels in HepG2 cells pretreated with MG132 (10 μM) for 4 h and then infected with CVB3 (MOI = 10) for 12 h.

c Western blot analysis of UBE4A levels in HepG2 cells pretreated with Methylamine (MA, 10 μM) for 4 h and then infected with CVB3 (MOI = 10) for 12 h.

d Western blot analysis of UBE4A levels in HepG2 cells transfected with increasing amount of Flag-3C (CVB3).

e RT-qPCR analysis of *Sgk1* mRNA in *Ube4a*$^{+/+}$ or *Ube4a*$^{-/-}$ cells transfected with Viperin, and then infected with or without CVB3 (MOI = 10) for 12 h.

NS, not significant ($p > 0.05$), one-way analysis of variance (ANOVA) (**a**), two-tailed unpaired Student's *t*-test (**e**). Data are shown as means ± SD of four biological replicates (**a, e**), or are representative of three independent experiments (**b**-**d**).

Supplementary Fig. S6

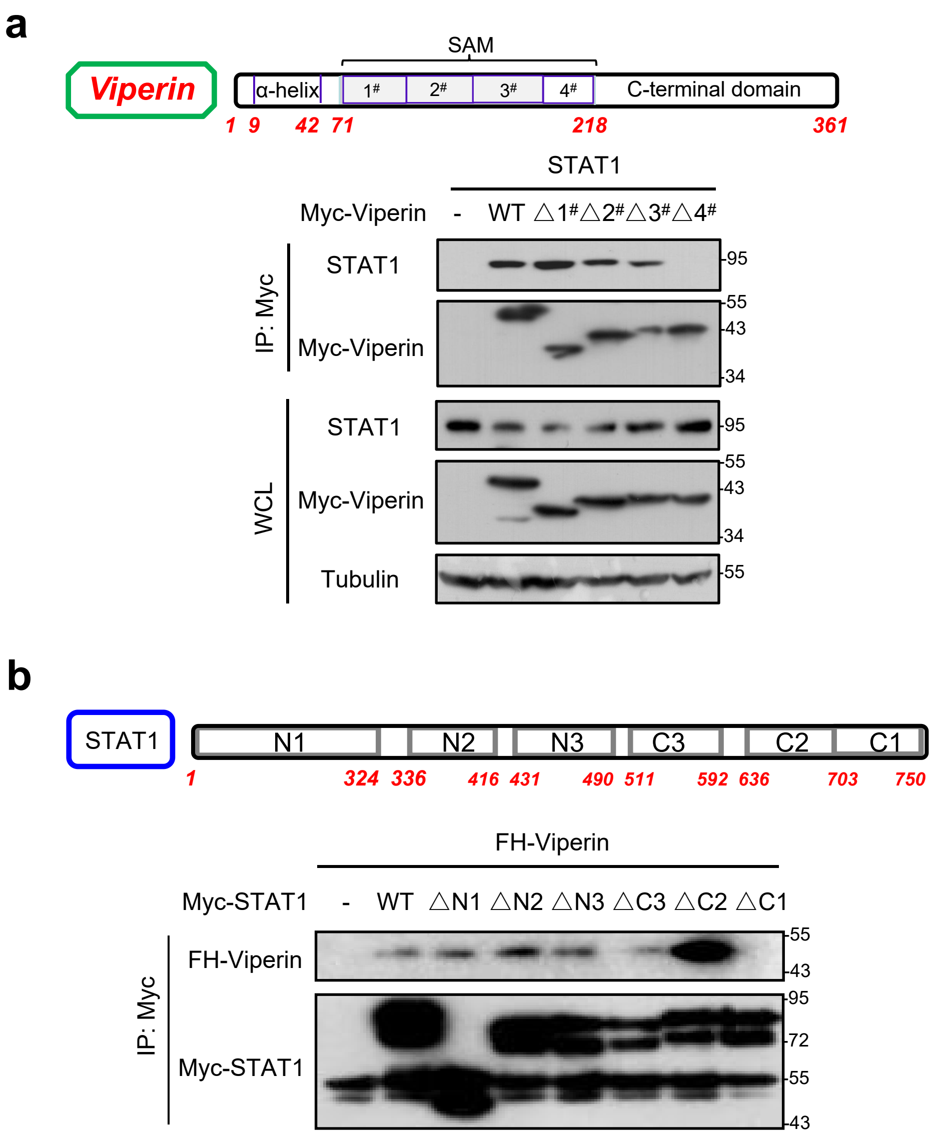

Supplementary Fig. S6. Mapping the interaction domain of STAT1 with Viperin.

a IP analysis of the interaction between Myc-Viperin (WT or deletion mutants) and STAT1 in HL-1 cells.

b IP analysis of the interaction between Flag-HA-Viperin (FH-Viperin) and Myc-STAT1 (WT or deletion mutants) in stable FH-Viperin-expressing HEK293T cells.

Data are representative of three independent experiments (**a, b**).

Supplementary Fig. S7

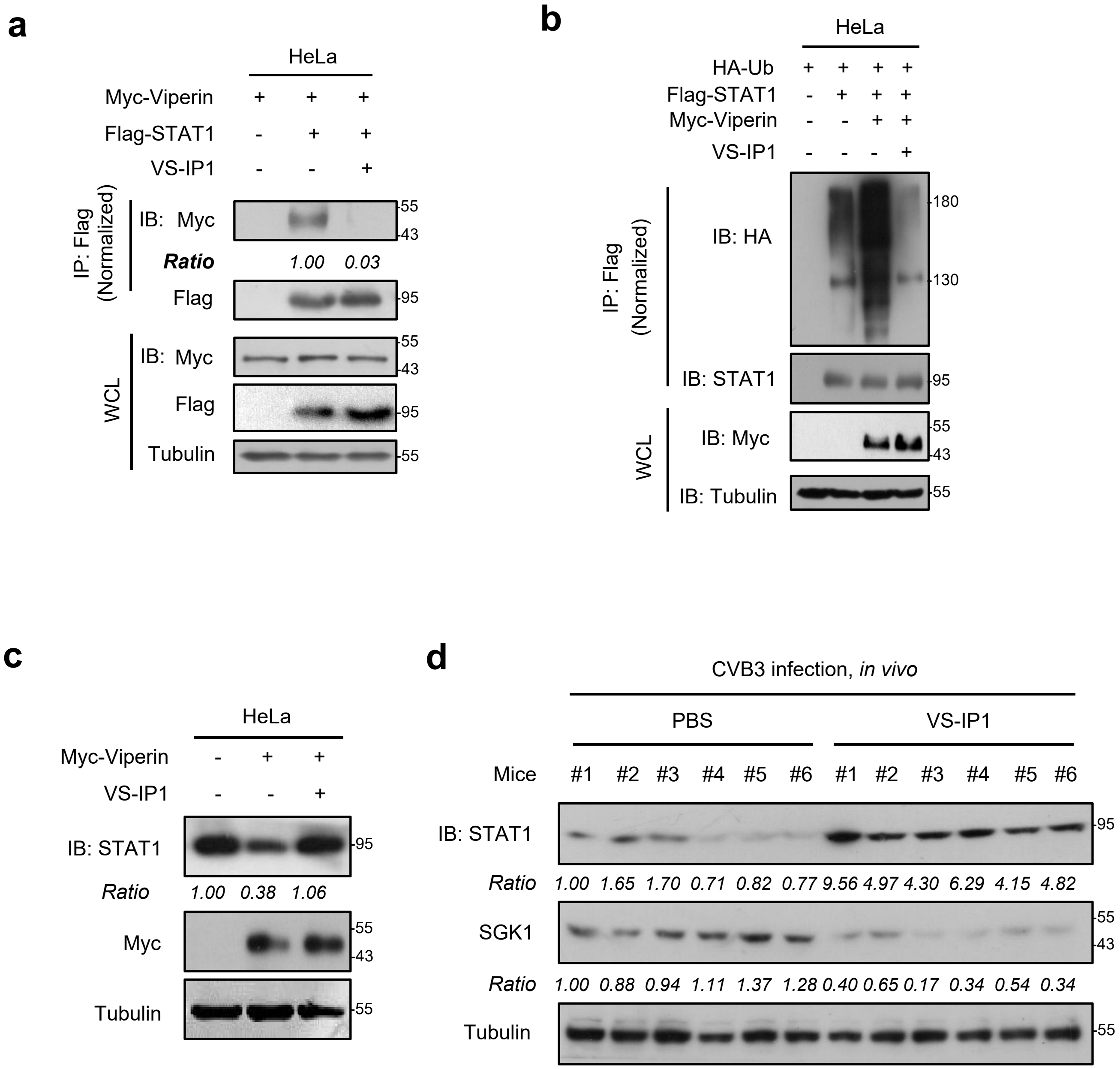

Supplementary Fig. S7. VS-IP1 inhibits the binding of Viperin with STAT1 and rescues STAT1 protein levels.

a IP analysis of the interaction between Myc-Viperin and Flag-STAT1 in HeLa cells transfected with Flag-STAT1 and Myc-Viperin and then treated with VS-IP1 (200 μM) for 12 h.

b IP analysis of Flag-STAT1 ubiquitination in HeLa cells transfected with Flag-STAT1, Myc-Viperin and HA-Ub, and then treated with VS-IP1 (200 μM) for 12 h.

c Western blot analysis of STAT1 levels in HeLa cells transfected with Myc-Viperin and then treated with VS-IP1 (200 μM) for 12 h.

d C57BL/6 mice were injected with PBS (n = 6, i.p.) or VS-IP1 (n = 6, i.p., 5 mg/kg) for 12 h and then infected with CVB3 ($2x10^5$ PFU per gram body, i.p.) for 7 days. STAT1 and SGK1 protein levels in mouse hearts were analyzed by western blot.

Data are representative of three independent experiments (**a**-**d**).
